# Supplementary material for: Regioselective stilbene O-methylations in Saccharinae grasses
Source: Nat Commun. 2023 Jun 12;14:3462. doi: 10.1038/s41467-023-38908-5 (PMC10261104; doi:10.1038/s41467-023-38908-5)
Supplement: Supplementary file 1 — Supplementary Information [file 41467_2023_38908_MOESM1_ESM.pdf]

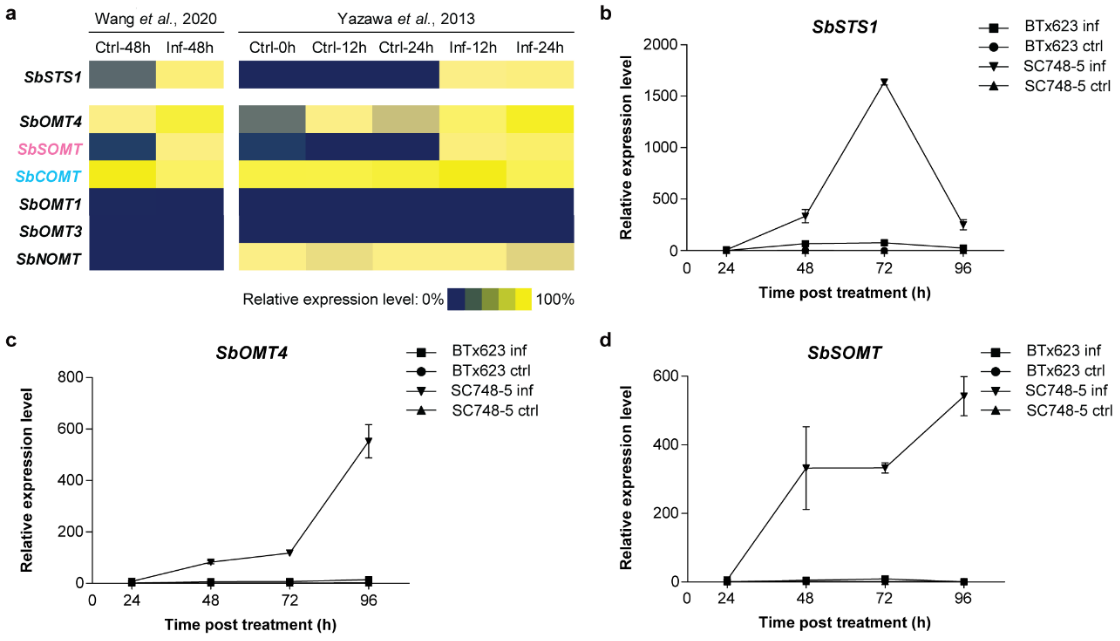

Supplementary Figure 1. Expression analysis of pterostilbene biosynthesis related candidate genes in sorghum.

a, *In silico* gene expression analysis. RNA-sequencing datasets derived from *Bipolaris sorghicola*-infected sorghum leaves and *Colletotrichum sublineola*-infected sorghum mesocotyls were obtained from literature[40,41].

b–d, Quantitative RT-PCR gene expression analysis of *SbSTS1* (**b**), *SbOMT4* (**c**) and *SbSOMT* (**d**). Expression levels are expressed relative to *Sorghum bicolor Eukaryotic Initiation Factor 4A-1* (*SbEIF4α*)[68]. Values refer to means ± SD (n = 3).

inf, infected; ctrl, control.

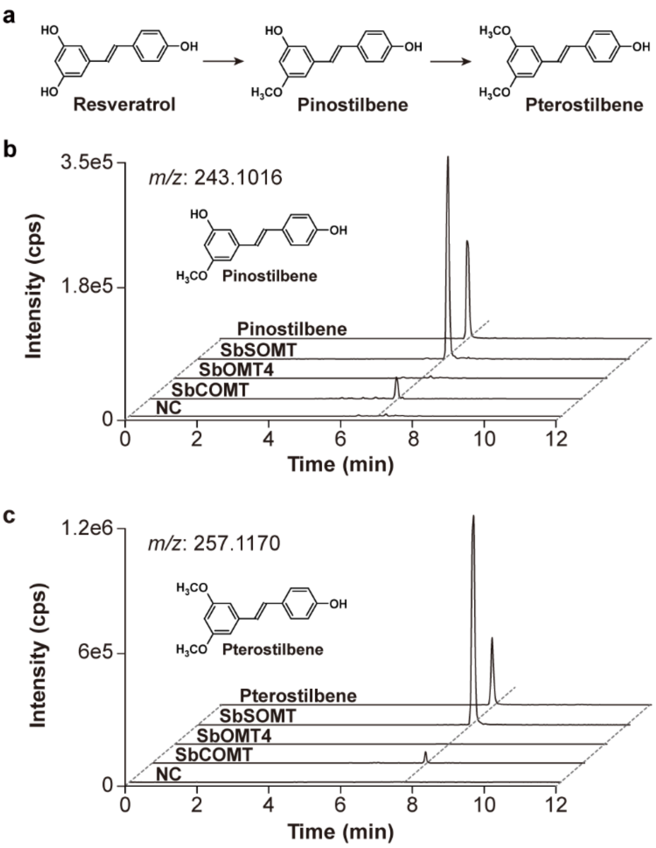

Supplementary Figure 2. Initial screening of catalytic activities of sorghum OMTs.

a, *In vitro O*-methylation of resveratrol by sorghum OMTs.

b-c, HPLC-QTOF-HRMS detection of pinostilbene (**b**), and pterostilbene (**c**) in sorghum OMTs catalyzed reaction using resveratrol as a substrate.

cps, counts per second; NC, negative control (reaction without enzyme).

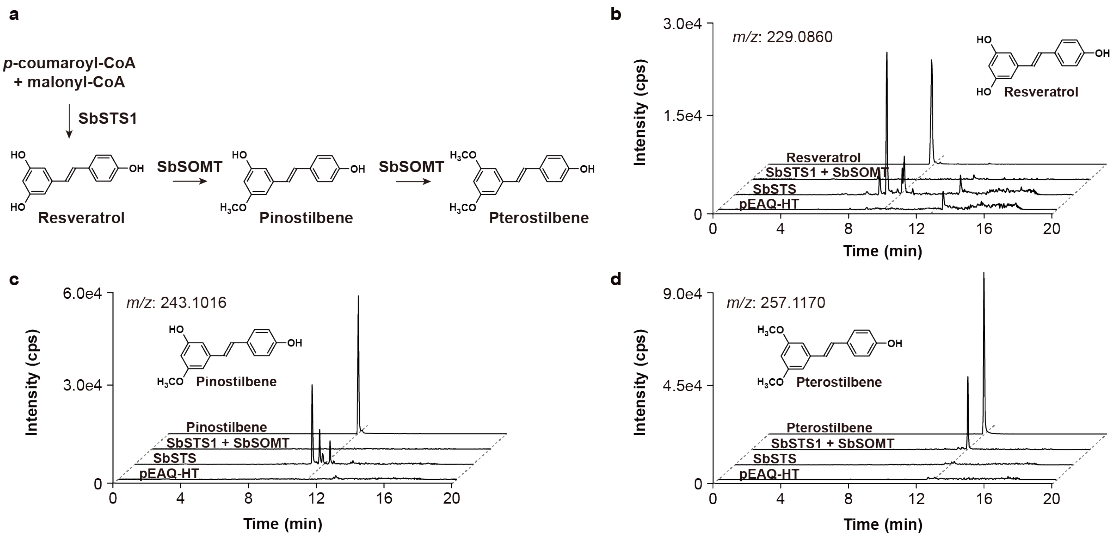

Supplementary Figure 3. Co-overexpression of *SbSTS1* and *SbSOMT* in *Nicotiana benthamiana* leaves.

a, *In planta* generation of pterostilbene by sorghum SbSTS1 and SbSOMT.

b-d, HPLC-QTOF-HRMS detection of resveratrol (**b**), pinostilbene (**c**), and pterostilbene (**d**) in *Nicotiana benthamiana* leaves transiently expressing *SbSTS1* or co-expressing *SbSTS1* and *SbSOMT*.

cps, counts per second.

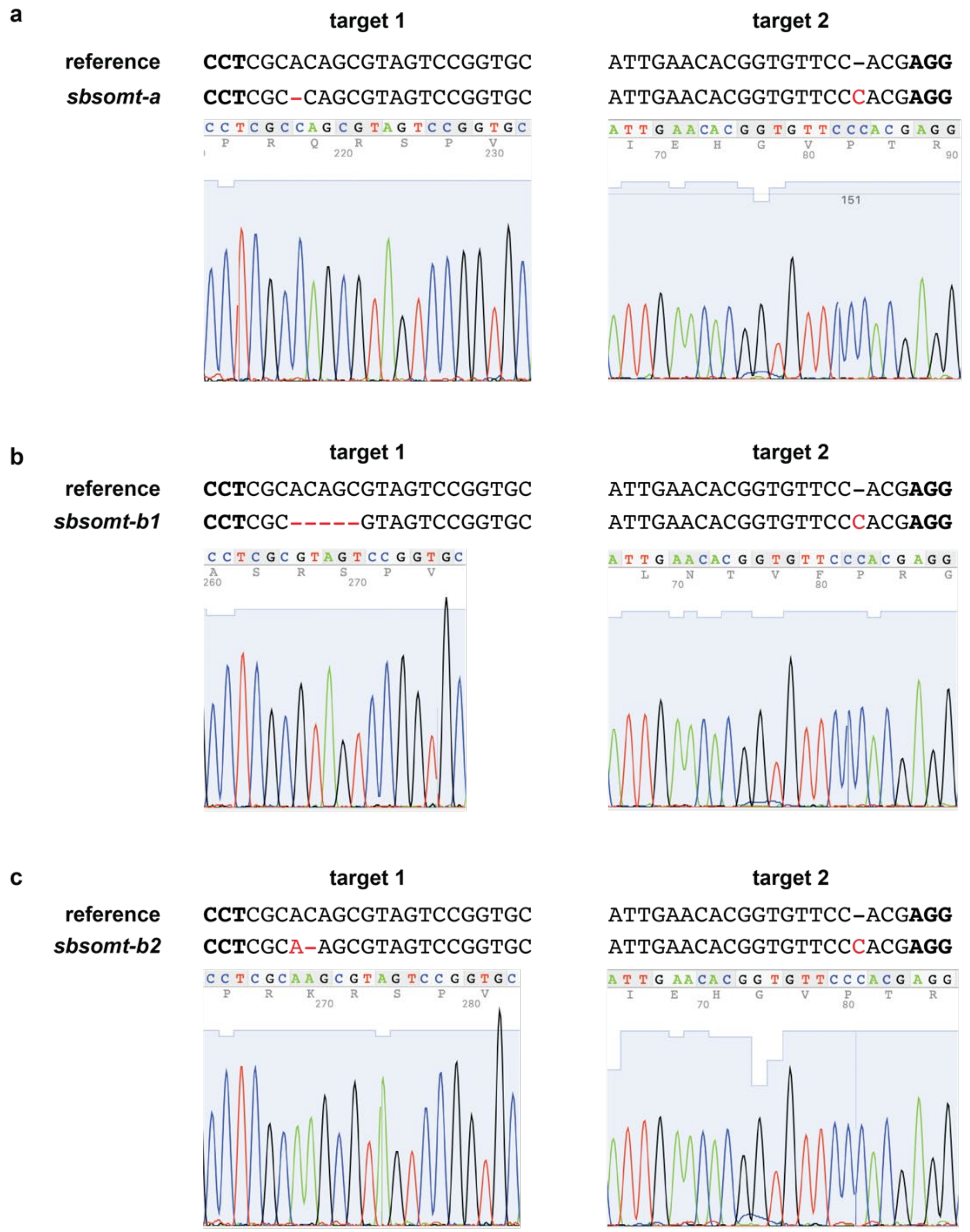

Supplementary Figure 4. **Genotyping of sorghum *sbsomt* mutants.**

a-c, Representative chromatographs of direct sequencing of the target sites in the *sbsomt-a* (**a**), *sbsomt-b1* (**b**), and *sbsomt-b2* (**c**) mutant lines (T_1 generation) are shown.

In red, deletion or insertion. Bold, protospacer adjacent motif (PAM) site.

>SbSOMT

```
WT          MGSYDSSSSSSNDSSARNEEDESCMFALKLLGGFAVPFTIKAVIELGVMDQLLTAERAMS     60
sbsomt-a    MGSYDSSSSSSNDSSARNEEDESCMFALKLLGGFAVPFTIKAVIELGVMDQLLTAERAMS     60
sbsomt-b1   MGSYDSSSSSSNDSSARNEEDESCMFALKLLGGFAVPFTIKAVIELGVMDQLLTAERAMS     60
sbsomt-b2   MGSYDSSSSSSNDSSARNEEDESCMFALKLLGGFAVPFTIKAVIELGVMDQLLTAERAMS     60

WT          AEELVAAAVAAQLPRPEVACTMVDRLLRFLASHSVVRCTTEVVVGTDD------------    108
sbsomt-a    AEELVAAAVAAQLPRPEVACTMVDRLLRFLASPA*SGARPRWWWARTTP--PPPPAAAGA    117
sbsomt-b1   AEELVAAAVAAQLPRPEVACTMVDRLLRFLASRSPVHDRGGGGHGRRHHHHLLPPELRRV    120
sbsomt-b2   AEELVAAAVAAQLPRPEVACTMVDRLLRFLASQA*SGARPRWWWARTTP--PPPPAAAGA    117

WT          -------------ATTTTCCRRSYAASPVCKWFARNGVEDSVLPLGMMILNKTFLDSWQN    155
sbsomt-a    TPRHPSASGSPGTASRIRCFRLG**S*TRHSWTAGKT---------------LRMQCWKE    159
sbsomt-b1   TRL----------QVVRQERRRGFG-----------------------------ASAWDD    141
sbsomt-b2   TPRHPSASGSPGTASRIRCFRLG**S*TRHSWTAGKT---------------LRMQCWKE    159

WT          ITDAVLEGAAPFEKTYGMPMFEYLSTNGPLNT----------------------------    187
sbsomt-a    QH--------HLRKPTGCQCSST*VQTDH*TR----------------------------    181
sbsomt-b1   DPKQDIP--GQLAKHYGCSVGRSSTI*ENLRDANVRVPKYKRTIEHGVPTRQWQIIR*L*    196
sbsomt-b2   QH--------HLRKPTGCQCSST*VQTDH*TR----------------------------    181

WT          ---VFHEAMANHSMI-ITKKLLKFFRGFEGLDVLVDVGGGNGTTLQMIRGQYKNMRGINY    243
sbsomt-a    ---CSHEAMANHSMI-ITKKLLKFFRGFEGLDVLVDVGGGNGTTLQMIRGQYKNMRGINY    237
sbsomt-b1   PRNCSSSSAASKALMCWST*AAATAPRCK*LEVNIRI*EA*TTTFLMSLRRLHQLKVWNM    252
sbsomt-b2   ---CSHEAMANHSMI-ITKKLLKFFRGFEGLDVLVDVGGGNGTTLQMIRGQYKNMRGINY    237

WT          DLPH--VIAQ------------------------AAPVEGVEHVGGSMFDNIPRGN----    273
sbsomt-a    DLPH--VIAQ------------------------AAPVEGVEHVGGSMFDNIPRGN----    267
sbsomt-b1   *VAACSIIFHAEMQFCSSGFFMIGTTRRASRS*RIAIQLSM*EAR*SFWSTLFRMNQNLL    308
sbsomt-b2   DLPH--VIAQ------------------------AAPVEGVEHVGGSMFDNIPRGN----    267

WT          ---AVLLKWILHDWDDKACIKILKNCYTALHVRGKVIVLEYVVPDEPEPTLAAQGAFELD    330
sbsomt-a    ---AVLLKWILHDWDDKACIKILKNCYTALHVRGKVIVLEYVVPDEPEPTLAAQGAFELD    324
sbsomt-b1   LQLRVPSNWTSPCWSRLAVVKRGH--------RGSS-------PSSPW---RPASLESLR    350
sbsomt-b2   ---AVLLKWILHDWDDKACIKILKNCYTALHVRGKVIVLEYVVPDEPEPTLAAQGAFELD    324

WT          LTMLVTFGSGKERTQREFSELAMEAGFSREFKATYIFANVWALEFTK*    377
sbsomt-a    LTMLVTFGSGKERTQREFSELAMEAGFSREFKATYIFANVWALEFTK*    371
sbsomt-b1   LRISLP-TSGPLSSQS--------------------------------    365
sbsomt-b2   LTMLVTFGSGKERTQREFSELAMEAGFSREFKATYIFANVWALEFTK*    371
```

Supplementary Figure 5. Predicted effects of mutations in SbSOMT in sorghum *sbsomt* mutant lines.

The amino acid sequences of wild-type and mutant SbSOMT were aligned using Clustal Omega[88]. Sequence that is different from wild-type sequence is highlighted in blue. First premature stop codons are highlighted in orange. First methionine after the mutation sites is highlighted in pink. The catalytic residues of sorghum SbSOMT are highlighted in red and bolded. Amino acid residues contributing to the hydrophobic interactions with stilbene substrates are highlighted in purple and bolded.

```
SbSOMT      MGSYDSSSSSSNDSSARNEEDESCMFALKLLGGFAVPFTIKAVIELGVMDQLLTAERAMS    60
VvROMT      ---MDL-----ANGVISAELLHAQAHVWNHIFNFIKSMSLKCAIQLGIPDTIHNHGKPMT    52
PsPMT2      ----------MNMQSVKD-EEALRASALGLAFSLETPFLLKCAIRLKIPDIISKAGPD--    47
                                      .     .:   : :*..*.* : * : .

SbSOMT      AEELVAAAVAAQLPRPEVACTMVDRLLRFLASHSVVRCTTEVVVGTDDATTTTCCRRSYA    120
VvROMT      LPELV-----AKLPVHPKRSQCVYRLMRILVHSGFLAAQRV-QQGKEE--------EGYV    98
PsPMT2      -VSLSVHQIAAQLPSEDPDMGALSRILTYLSTMGILQAIVP----PEGVNAPMN--IRYG    100
              .*      *:**        : *::  *   ..: .        :           *

SbSOMT      ASPV-CKWFAR-NGVEDSVLPLGMMILNKTFLDSWQNITDAVL-EGAAPFEKTYG--MPM    175
VvROMT      LTDASRLLLM---DDSLSIRPLVLAMLDPILTKPWHYLSAWFQNDDPTPFHTTYE--RSF    153
PsPMT2      LTNLTKTYFTSEDISSRSLVPFVLLQTHPLYVTAWDNIHERVL-HGGDNFKNSSGNGKDF    159
             :      :      . *: *: :   .      *. :   .  ..   *..:      :

SbSOMT      FEYLSTNGPLNTVFHEAMANHSMIITKKLLKFFR-GFEGLDVLVDVGGGNGTTLQMIRGQ    234
VvROMT      WDYAGHEPQLNNSFNEAMASDARLLTSVLLKEGQGVFAGLNSLVDVGGGTGKVAKAIANA    213
PsPMT2      WNFAAGEPEFNAIFNAGMVSVTKATITYVLAVYD-GFKDINTLVDVGGGRGEALSLITEA    218
            ::: . :  :*  *: .*.. :    . :*      * .:: ******* * . . *

SbSOMT      YKNMRGINYDLPHVIAQAAPVEGVEHVGGSMFDNIPRGNAVLLKWILHDWDDKACIKILK    294
VvROMT      FPHLNCTVLDLPHVVAGLQGSKNLNYFAGDMFEAIPPADAILLKWTLHDWSDEECVKILK    273
PsPMT2      HPHIRAINFDLPQVIATAPTIPGVQHMSGNLFESAPSADAIFMKNFLHSWNDEDCIKLLN    278
            . ::.    ***:*:*      .::::..*.:*: * .:*:::*  **.*.*: *:*:*:

SbSOMT      NCYTALHVR---GKVIVLEYVVPDEPEPTLAAQGAFELDLTMLVTFGSGKERTQREFSEL    351
VvROMT      RCREAIPSKENGGKVIIIDMIMMKNQGDYKSIETQLFFDMTMM-IFAPGRERDENEWEKL    332
PsPMT2      NCHQALPEK---GKLILSEAILDLTEGSD-MIGSANVLDAVMLNCLPGGGERTRKQWNDL    334
            .*  *:  :   **:*: : ::               :* .*:  :  * ** ..::..*

SbSOMT      AMEAGFSREFKATYIFANVWALEFTK-    377
VvROMT      FLGAGFSHYKITPILGLRS-LIEVYP-    357
PsPMT2      LQAAGFSISKIVGRNGTLTKVIEAIKS    361
               ****    .         :*
```

Supplementary Figure 6. Multiple sequence alignment of *bona fide* stilbene *O*-methyltransferases (SOMTs).

The amino acid sequence of SbSOMT, VvROMT and PsPMT2 were aligned using Clustal Omega[88]. The catalytic residues of sorghum SbSOMT are colored in red and bolded, while those of other SOMTs are also colored in red. Amino acid residues contributing to the hydrophobic interactions with stilbene substrates are highlighted in purple and bolded. Fully conserved amino acid residues across all three SOMTs are indicated by asterisks; those with strongly similar properties are indicated with colons; those with weakly similar properties are indicated with periods.

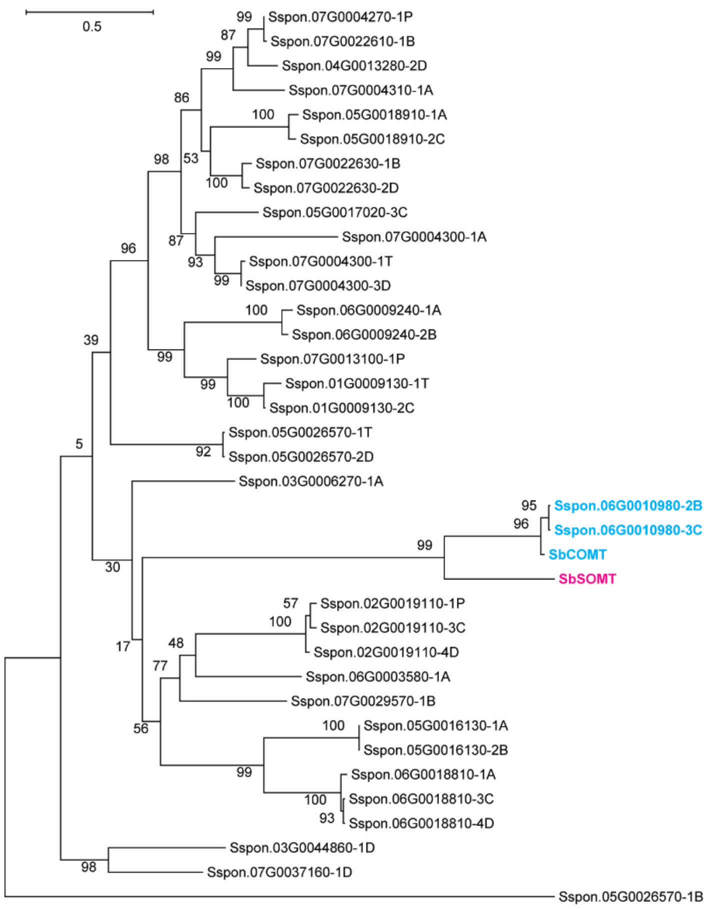

Supplementary Figure 7. Phylogeny of wild sugarcane OMTs.

Phylogenetic analysis of wild sugarcane OMTs. The unrooted phylogenetic tree was constructed by maximum likelihood using MEGA X[90]. Bootstrapping with 1,000 replications was carried out. Scale bar denotes 0.5 amino acid substitution per site.

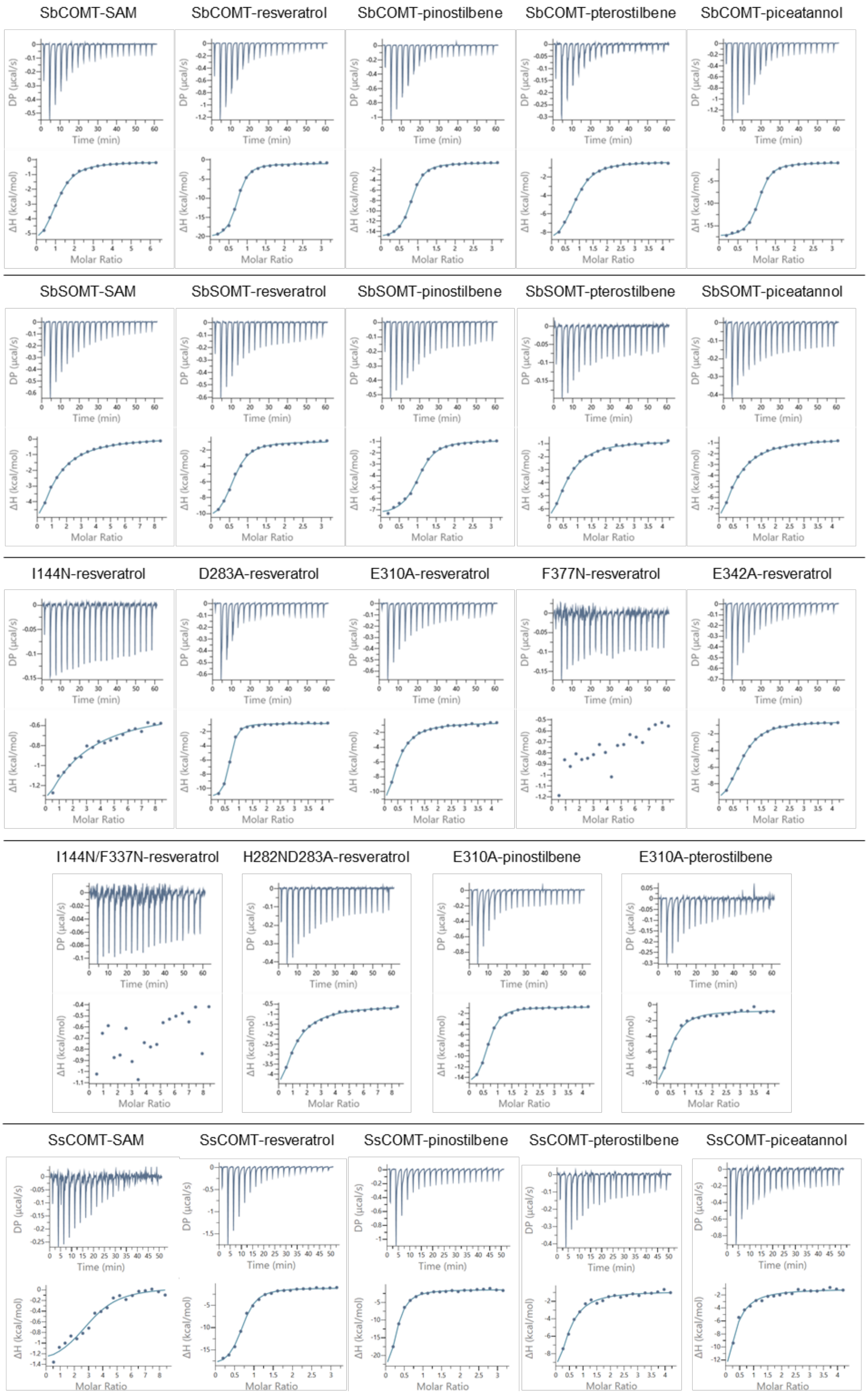

Supplementary Figure 8. Titration thermograms and fitting curves of conducted ITCs.

Each set of thermogram (above) and fitting curve (below) represents performed ITC of different combinations of protein (in cell) and ligand (in syringe) as titled.

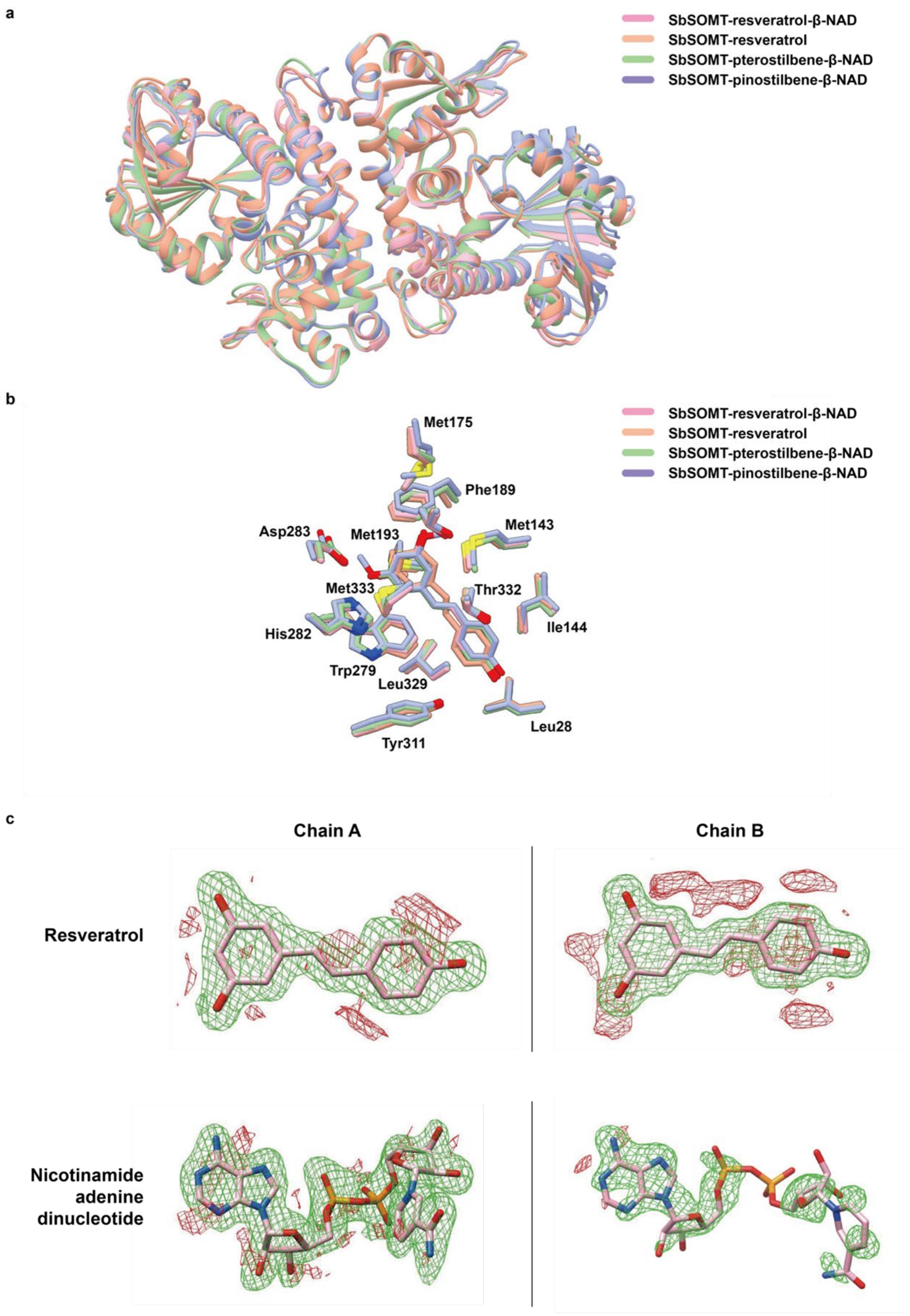

Supplementary Figure 9. Structures obtained in this study.

a, Superimposition of all four SbSOMT complexes obtained in this study. Superimposition was in reference to SbSOMT-resveratrol-β-NAD (pink) and revealed high similarity towards all SbSOMT-resveratrol (orange, RMSD = 0.468 Å), SbSOMT-pinostilbene-β-NAD (green, RMSD = 0.995 Å) and SbSOMT-pterostilbene-β-NAD

(purple, RMSD = 0.730 Å) complexes.

b, Close-up view on the superimposed stilbene derivatives observed in the solved structures and correspondent surrounding residues. Circled L28 is constituted in adjacent protomer.

c, The $F_O - F_C$ electron density omit maps of resveratrol and β-NAD ligands in SbSOMT-resveratrol-β-NAD ternary complex contoured at 3σ (green mesh) and -3σ (red mesh).

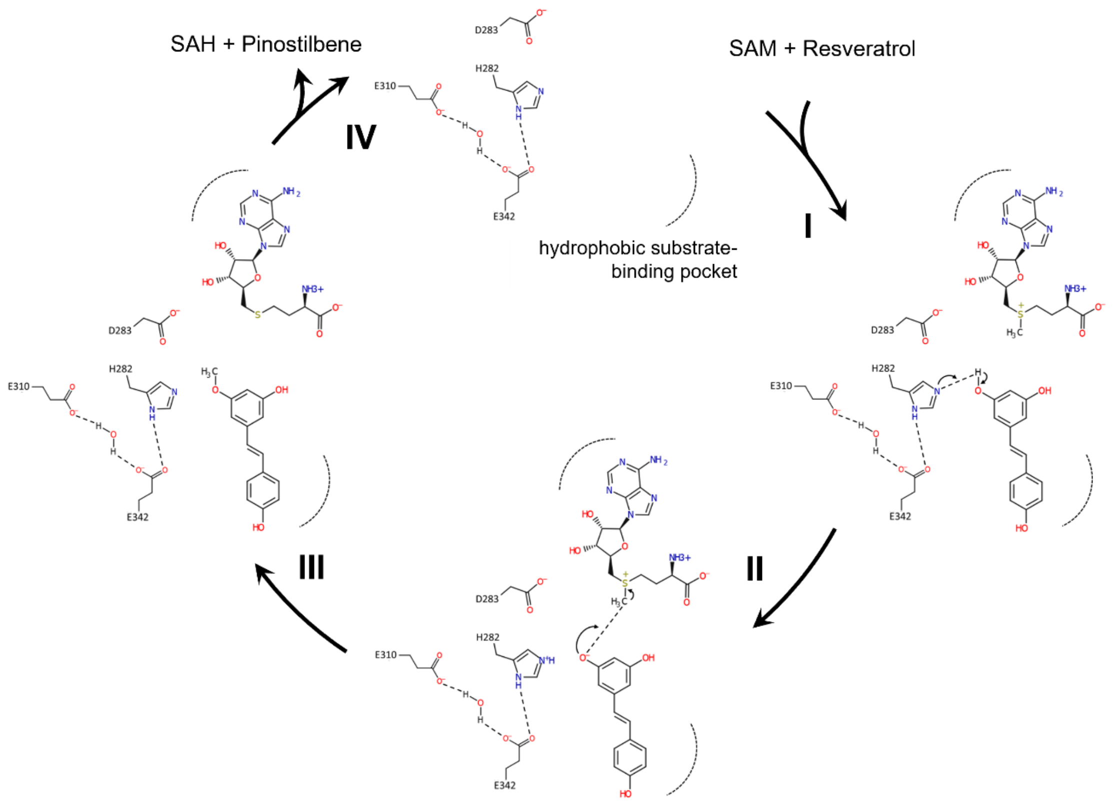

Supplementary Figure 10. Reaction mechanism of consecutive stilbene *O*-methylations mediated by SbSOMT.

Schematic of SbSOMT *O*-methylation cycle exemplified with *S*-adenosyl methionine (SAM, donor) and resveratrol (acceptor)[21,22,43,44]. Upon binding of SAM and resveratrol (Step I), the surrounding electrostatic interactions allow His282 N_ε to deprotonate 3-OH of resveratrol. Step II, deprotonated O^- catalyzes SN_2 attack towards methyl group of SAM sulfonium ylide to produce pinostilbene and *S*-adenosyl-homocysteine (SAH). Step III, the His282 protonated imidazole is regenerated by solvent and returns to the neutral state. Step IV, the SAH and pinostilbene are dissociated from SbSOMT.

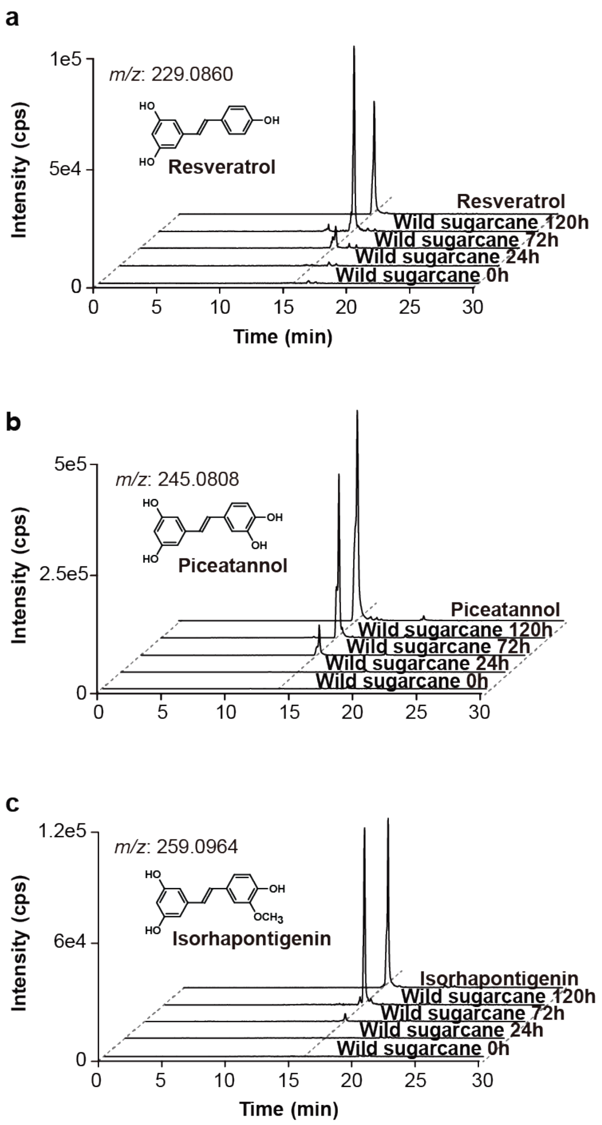

Supplementary Figure 11. Stilbene profiles of mechanically-wounded wild sugarcane stalks.

a-c, HPLC-QTOF-HRMS detection of resveratrol (**a**), piceatannol (**b**), and isorhapontigenin (**c**) in mechanically-wounded wild sugarcane stalks 120 h after treatment.

cps, counts per second.

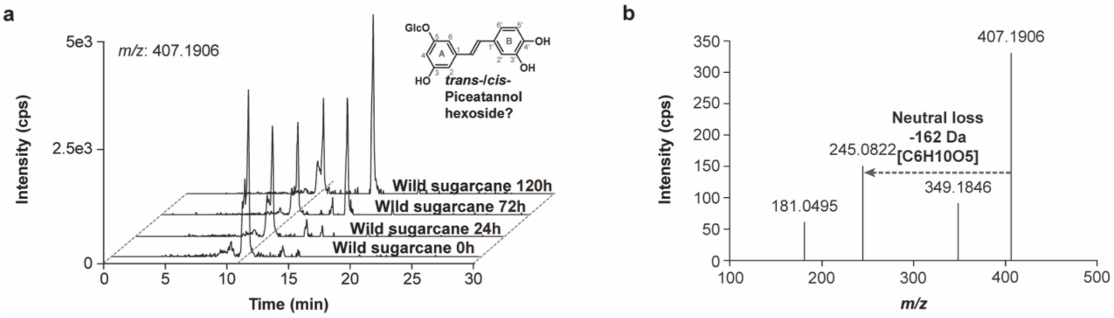

Supplementary Figure 12. Tentative identification of piceatannol-hexoside from mechanically wounded wild sugarcane stalks.

a, HPLC-QTOF-HRMS detection of putative piceatannol-hexoside.

b, MS^2 fragmentation pattern of putative piceatannol-hexoside. A neutral loss of 162 Da, potentially corresponding to an anhydrohexose unit, was observed for this ion.

Glc: glucose; cps, counts per second.

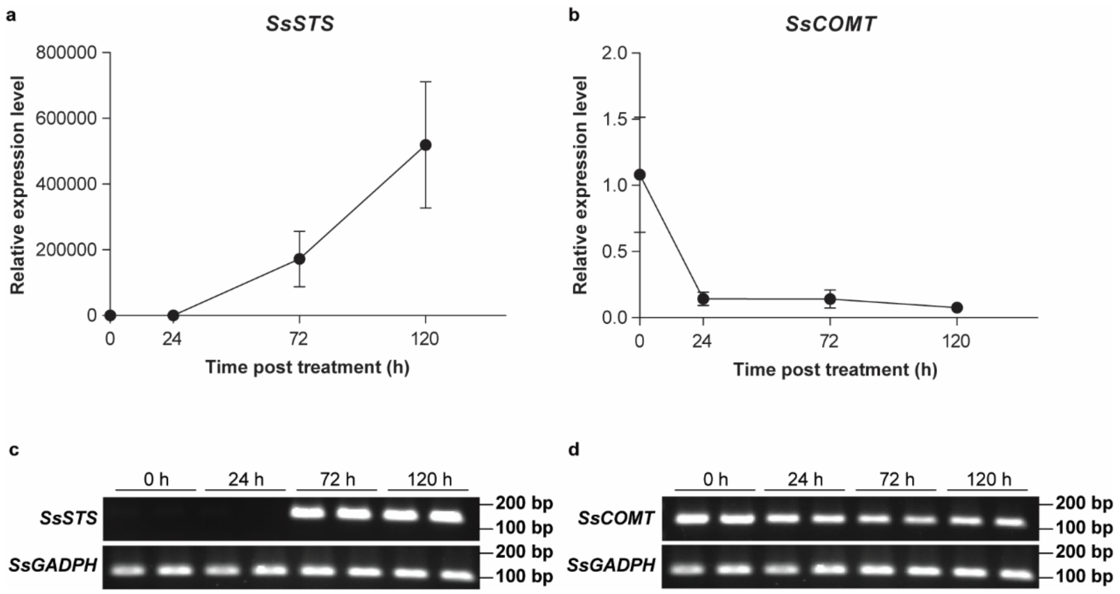

Supplementary Figure 13. Expression analysis of stilbene biosynthesis related genes in wild sugarcane.

a–b, Quantitative RT-PCR gene expression analysis of *SsSTS* (**a**) and *SsCOMT* (**b**). Expression levels are expressed relative to *Saccharum spontaneum Glyceraldehyde 3-Phosphate Dehydrogenase* (*SsGADPH*)[68]. Values refer to means ± SD (n = 5).

c-d, Semi-quantitative RT-PCR gene expression analysis of *SsSTS* (**c**) and *SsCOMT* (**d**) relative to *SsGADPH*. Thirty cycles were used for amplification. Two biological duplicates from each time point were analyzed. DNA marker used was labelled accordingly.

```
SbSTS1      MTTGKVTLEAVRKAQRAEGPATVLAIGTATPANCVYQADYPDYYFRVTKSEHLTDLKEKF      60
SsSTS       -MTGKVTLGAVRKAQRAEGSAAVLAIGTATPANCVYQADYPDYYFRVTKSEHLTDLKEKF      59
              ****** ********** *:**************************************

SbSTS1      KRICHKSMIRKRYMHLTEDILEENPNMSSYWAPSLDARQDILIQEIPKLGAEAAEKALKE      120
SsSTS       KRICHKSMITKRYMHLTEGFLQENPNMSSYSAPSLDARQDILIEEVPKLGAAAAEKALKE      119
            ********* ********.:*:******** ************:*:***** ********

SbSTS1      WGQPRSRITHLVFCTTSGVDMPGADYQLIKLLGLCPSVNRAMMYHQGCFAGGMVLRLAKD      180
SsSTS       WGQPRSQITHLVFCTTSGVDMPGADYQLIKLLGLSLSVNRAMMYHQGCFAGGMVLRLAKD      179
            ******:***************************. ************************

SbSTS1      LAENNRGARVLIVCSEITVVTFRGPSESHLDSLVGQALFGDGAAAVIVGADPSEPAERPL      240
SsSTS       LAENNRGARVLIVCSEITAVTFRGPSESHLDSLVGQALFGDGAAAVIVGADPSA-AEWPL      238
            ******************.**********************************  ** **

SbSTS1      FHLVSASQTILPDSEGAIEGHLREVGLTFHLQDRVPQLISMNIERLLEDAFAPLGISDWN      300
SsSTS       FQLVSASQTILPDSEGAIEGHLREVGLTFHLQDRVPQLISTNIERLLEDAFTPLGISDWN      298
            *:************************************** **********:********

SbSTS1      SIFWVAHPGGPAILNMVEAKVGLDKARMCATRHILAEYGNMSSVCVLFILDEMRNRSAKD      360
SsSTS       SIFWVAHPGGPAILNMVEAKAGLDKARLCATRHILAEYGNMSSACVLFILDEMRNKSAED      358
            ********************.******:***************.***********:**:*

SbSTS1      GHTTTGEGMEWGVLFGFGPGLTVETIVLHSVPITTVAA*     398
SsSTS       GHTTTGEGMEWGVLFGFGPGLTVETIVLQSVPIITV*--     394
            ****************************:**** **
```

Supplementary Figure 14. Multiple sequence alignment of SbSTS1 and SsSTS.

The amino acid sequences of SbSTS1 and SsSTS were aligned using Clustal Omega[88]. Fully conserved amino acid residues are indicated by asterisks; those with strongly similar properties are indicated with colons; those with weakly similar properties are indicated with periods.

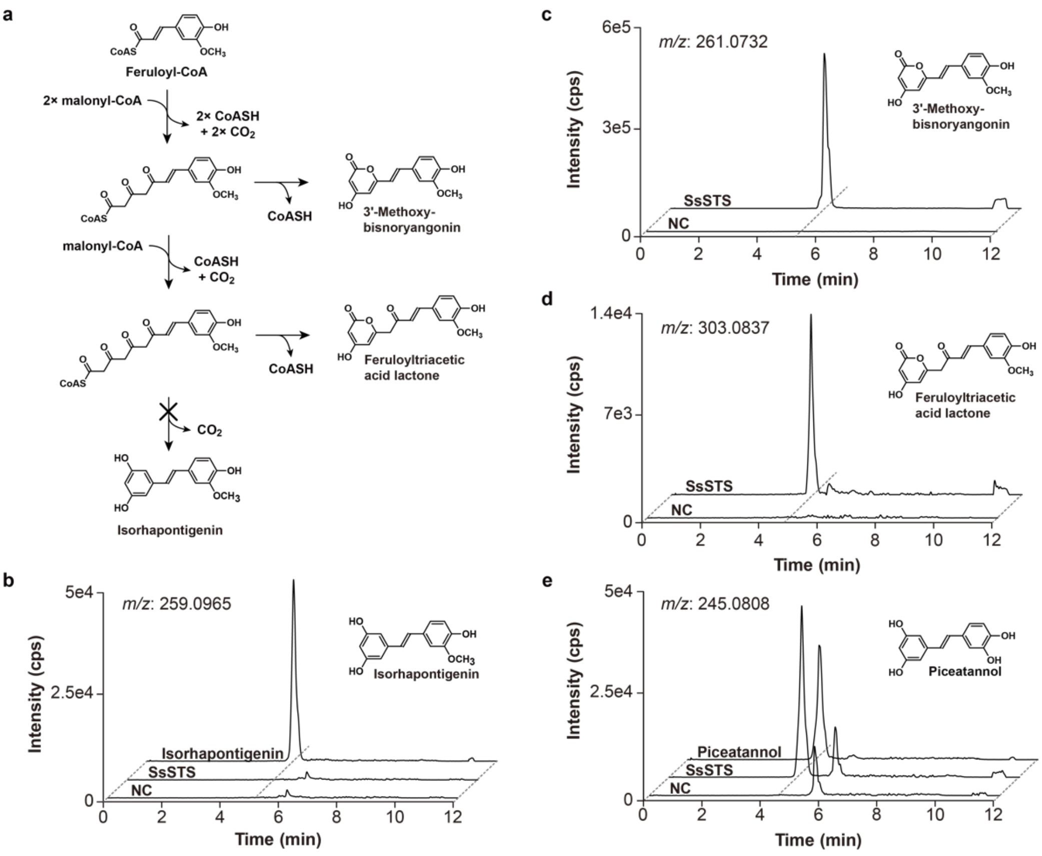

Supplementary Figure 15. Catalytic activities of SsSTS.

a, Proposed STS reaction with feruloyl-CoA and malonyl-CoA as substrates.

b–d, HPLC-QTOF-HRMS detection of isorhapontigenin (**b**), 3′-methoxybisnoryangonin (**c**), and feruloyltriacetic acid lactone (**d**) in SsSTS catalyzed reaction using feruloyl-CoA and malonyl-CoA as substrates. The detection of these derailment products is consistent with a previous report showing that feruloyl-CoA is a poor substrate for STSs, at least under *in vitro* conditions[5].

e, HPLC-QTOF-HRMS detection of piceatannol in SsSTS catalyzed reaction using caffeoyl-CoA and malonyl-CoA as substrates.

cps, counts per second; NC, negative control (reaction without enzyme).

```
SbSOMT   MGSYDSSSSSSNDSSARNEEDESCMFALKLLGGFAVPFTIKAVIELGVMDQLLTAE---R   57
SbCOMT   -------MGSTAEDVAAVADEEACMYAMQLASSSILPMTLKNALELGLLEVLQKD--AGK   51
SsCOMT   -------MGSTAEDVAAVADEEACMYAMQLASASILPMTLKNALELGLLEVLQAEAPAGK   53
                 .*: :. *   ::*:**:*::*  ..  :*:*:* .:***::: *       :

SbSOMT   AMSAEELVAAAVAAQLPRPEVACTMVDRLLRFLASHSVVRCTTEVVVGTDDATTTTCCRR   117
SbCOMT   ALAAEEVVARL--PVAPTNPAAADMVDRMLRLLASYDVVKCQMEDKDG--------KYER   101
SsCOMT   ALAPEEVVARL--PVAPNNPDAADMVDRMLRLLASYDVVKCQMEDKDG--------KYER   103
         *:: **:**       *    *.  ****:**:***:.**:*  *   *          .*

SbSOMT   SYAASPVCKWFARNGVEDSVLPLGMMILNKTFLDSWQNITDAVLEGAAPFEKTYGMPMFE   177
SbCOMT   RYSAAPVGKWLTPNEDGVSMAALALMNQDKVLMESWYYLKDAVLDGGIPFNKAYGMTAFE   161
SsCOMT   RYSAAPVGKWLTPNEDGVSMAALTLMNQDKVLMESWYYLKDAVLDGGIPFNKAYGMTAFE   163
          *:*:** **:: *    *:   * :*   :*.:::**  :.****:*.  **:*:***  **

SbSOMT   YLSTNGPLNTVFHEAMANHSMIITKKLLKFFRGFE-GLDVLVDVGGGNGTTLQMIRGQYK   236
SbCOMT   YHGTDPRFNRVFNEGMKNHSVIITKKLLEFYTGFDESVSTLVDVGGGIGATLHAITSHHS   221
SsCOMT   YHGTDPRFNRVFNEGMKNHSVIITKKLLEFYTGFE-GVSTLVDVGGGIGATLHAITSHHS   222
         * .*:  :* **:*.* ***:*******:*: **: .:..******* *:**: * .::.

SbSOMT   NMRGINYDLPHVIAQAAPVEGVEHVGGSMFDNIPRGNAVLLKWILHDWDDKACIKILKNC   296
SbCOMT   HIRGVNFDLPHVISEAPPFPGVQHVGGDMFKSVPAGDAILMKWILHDWSDAHCATLLKNC   281
SsCOMT   QIKGINFDLPHVISEAPPFPGVQHVGGDMFKSVPAGDAILMKWILHDWSDAHCATLLKNC   282
         :::*:*:******::* *. **:****.**..:* *:*:*:*******.*  * .:****

SbSOMT   YTALHVR-GKVIVLEYVVPDEPEPTLAAQGAFELDLTMLVTFGSGKERTQREFSELAMEA   355
SbCOMT   YDALPEKGGKVIVVECVLPVTTDAVPKAQGVFHVDMIMLAHNPGGRERYEREFRDLAKAA   341
SsCOMT   YDALPEN-GKVIIVECVLPVNTEAVPKAQGVFHVDMIMLAHNPGGRERYEREFHDLAKGA   341
         * **  . ****::* *:*   : .  ***.*.:*: **.   .*:** :*** :**  *

SbSOMT   GFSREFKATYIFANVWALEFTK*   377
SbCOMT   GFS-GFKATYIYANAWAIEFIK*   362
SsCOMT   GFS-GFKATYIYANAWAIEFIK*   362
         ***  ******:**.**:** **
```

Supplementary Figure 16. Multiple sequence alignment of SbCOMT, SbSOMT, and SsCOMT.

The amino acid sequences of SbCOMT, SbSOMT, and SsCOMT were aligned using Clustal Omega[88]. Fully conserved amino acid residues are indicated by asterisks; those with strongly similar properties are indicated with colons; those with weakly similar properties are indicated with periods. Residues of SbSOMT (in pink) and SsCOMT (in blue) corresponding to SbCOMT Asn128 and Asn323 were highlighted and bolded.

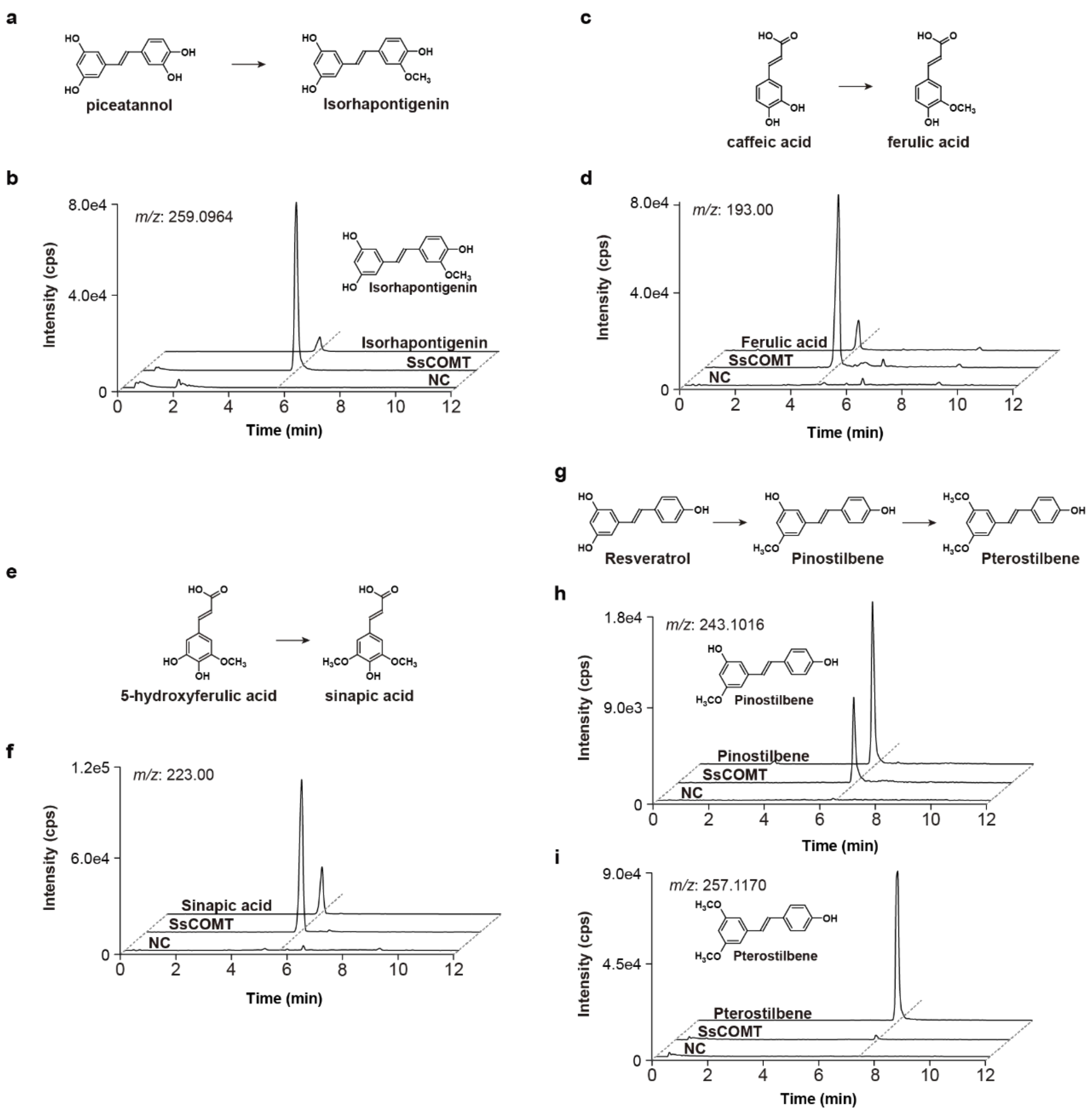

Supplementary Figure 17. Catalytic activities of SsCOMT.

a, *In vitro O*-methylation of piceatannol by SsCOMT.

b, High-performance liquid chromatography-tandem MS (HPLC-MS/MS) detection of isorhapontigenin in SsCOMT catalyzed reaction using resveratrol as a substrate.

c, *In vitro O*-methylation of caffeic acid by SsCOMT.

d, High-performance liquid chromatography-tandem MS (HPLC-MS/MS) detection of ferulic acid in SsCOMT catalyzed reaction using caffeic acid as a substrate.

e, *In vitro O*-methylation of 5-hydroxyferulic acid by SsCOMT.

f, High-performance liquid chromatography-tandem MS (HPLC-MS/MS) detection of sinapic acid in SsCOMT catalyzed reaction using 5-hydroxyferulic acid as a substrate.

g, *In vitro O*-methylation of resveratrol by SsCOMT.

h-i, High-performance liquid chromatography-tandem MS (HPLC-MS/MS) detection of pinostilbene (**b**), and pterostilbene (**c**) in SsCOMT catalyzed reaction using resveratrol as a substrate.

cps, counts per second; NC, negative control (reaction without enzyme).

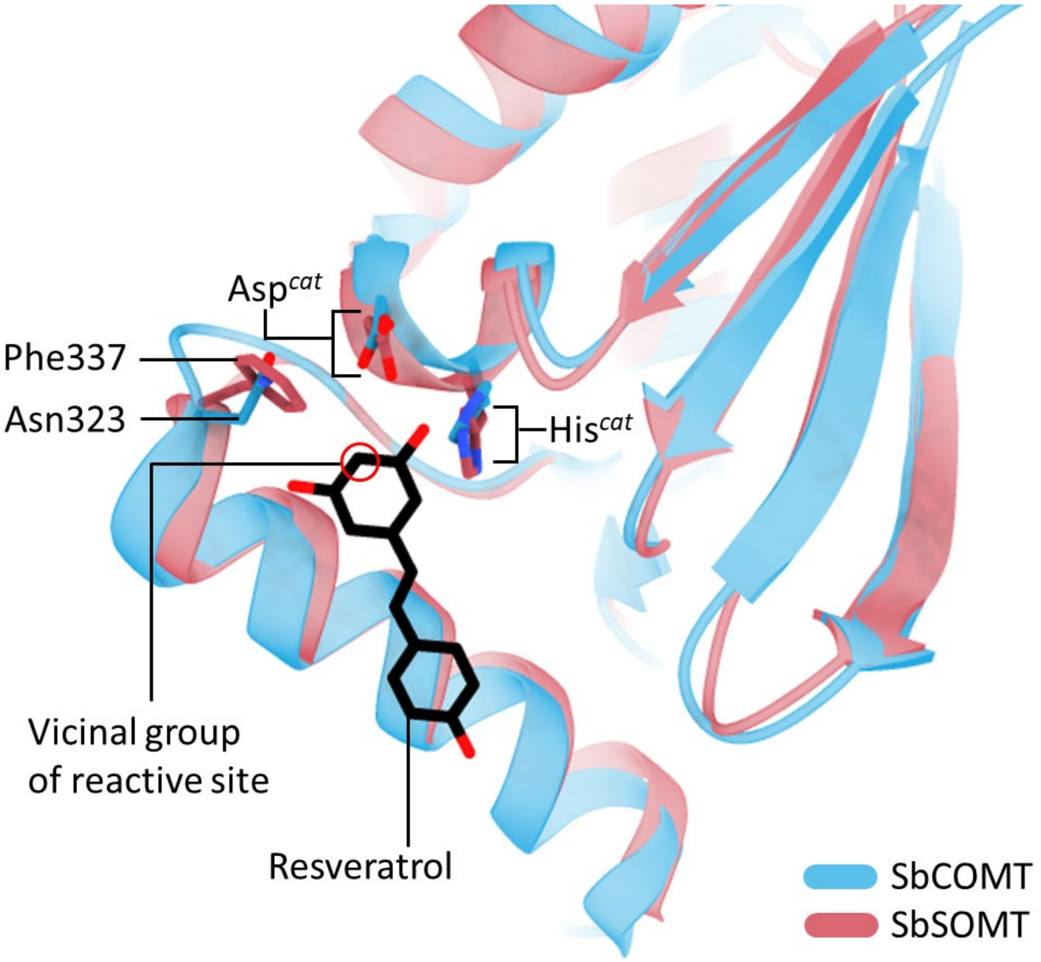

Supplementary Figure 18. Side view of SbCOMT^{Asn323}/SbSOMT^{Phe337}, catalytic dyad (His^{cat} and Asp^{cat}) and resveratrol of sorghum OMTs.

The position of SbCOMT^{Asn323}/SbSOMT^{Phe337} is at proximity to the catalytic dyads and hence contributes to substrate orientation via polarity pairing. The aromatic carbon (C4) vicinal to the methyl-accepting 3-OH of resveratrol is positioned in close proximity to Phe337 of SbSOMT.

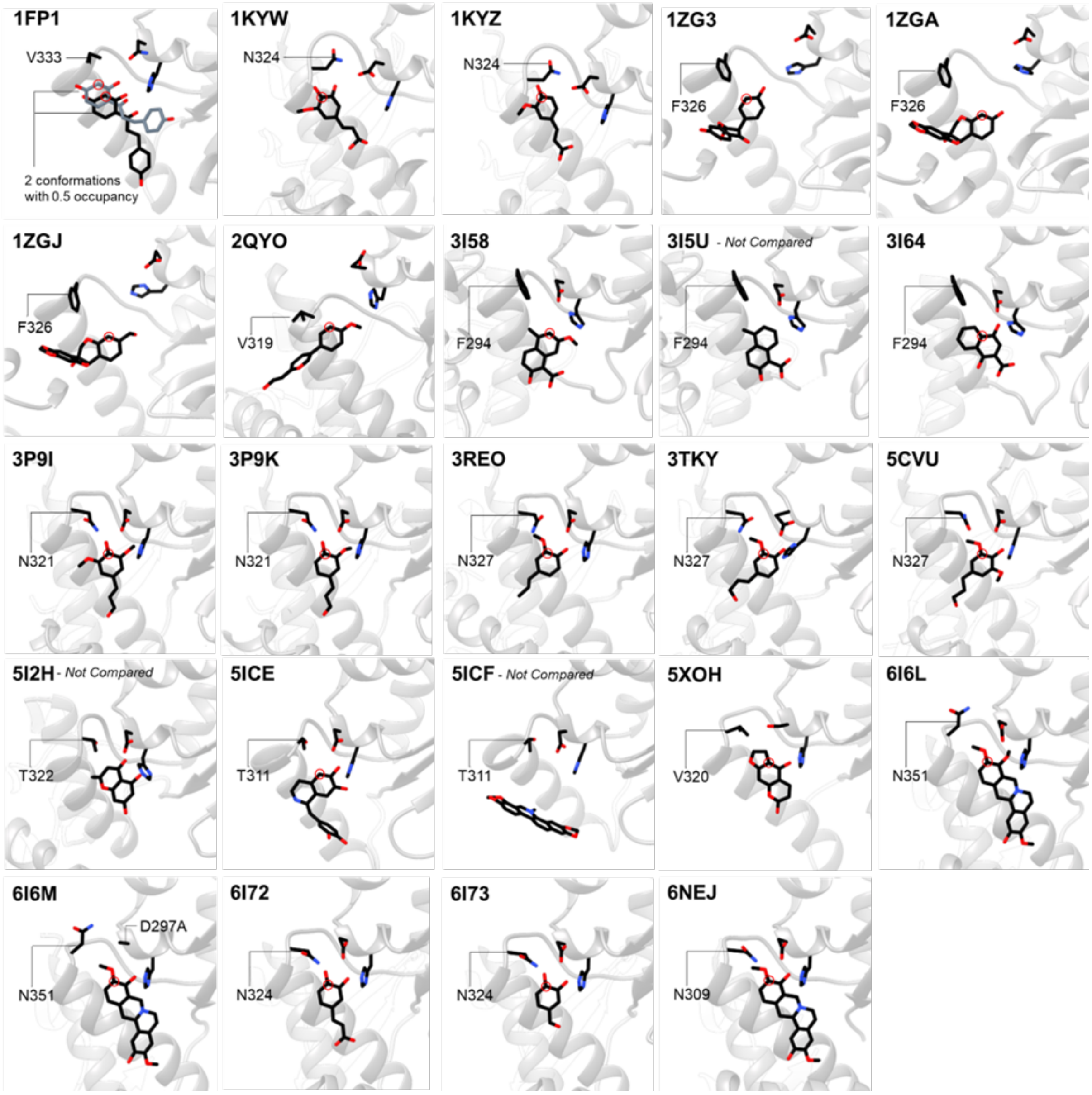

Supplementary Figure 19. Polarity pairing as a common regioselective mechanism across OMT-ligand complexes deposited to PDB.

Twenty-four unique OMT-ligand complexes were retrieved from PDB (by 10 August 2022), and their respective amino acid residue equivalent to SbCOMT^{Asn323}/ SbSOMT^{Phe337}, catalytic dyad, and bound ligand were shown. We retrieved via homolog search (≥30% identity to SbCOMT or SbSOMT) and screened for complexes that are bound to ligand at substrate binding pocket. Further details are listed in Supplementary Table **8**. Appropriate polarity pairing between this residue's side chain and the functional group vicinal to the reactive site (methyl-accepting -OH group) was observed in all complexes, except for the three structures excluded from our analysis, either due to the use of non-reactive ligand (3I5U and 5ICF) or non-productive OMT-ligand conformation (5I2H). The carbon vicinal to the reactive site is circled in red.

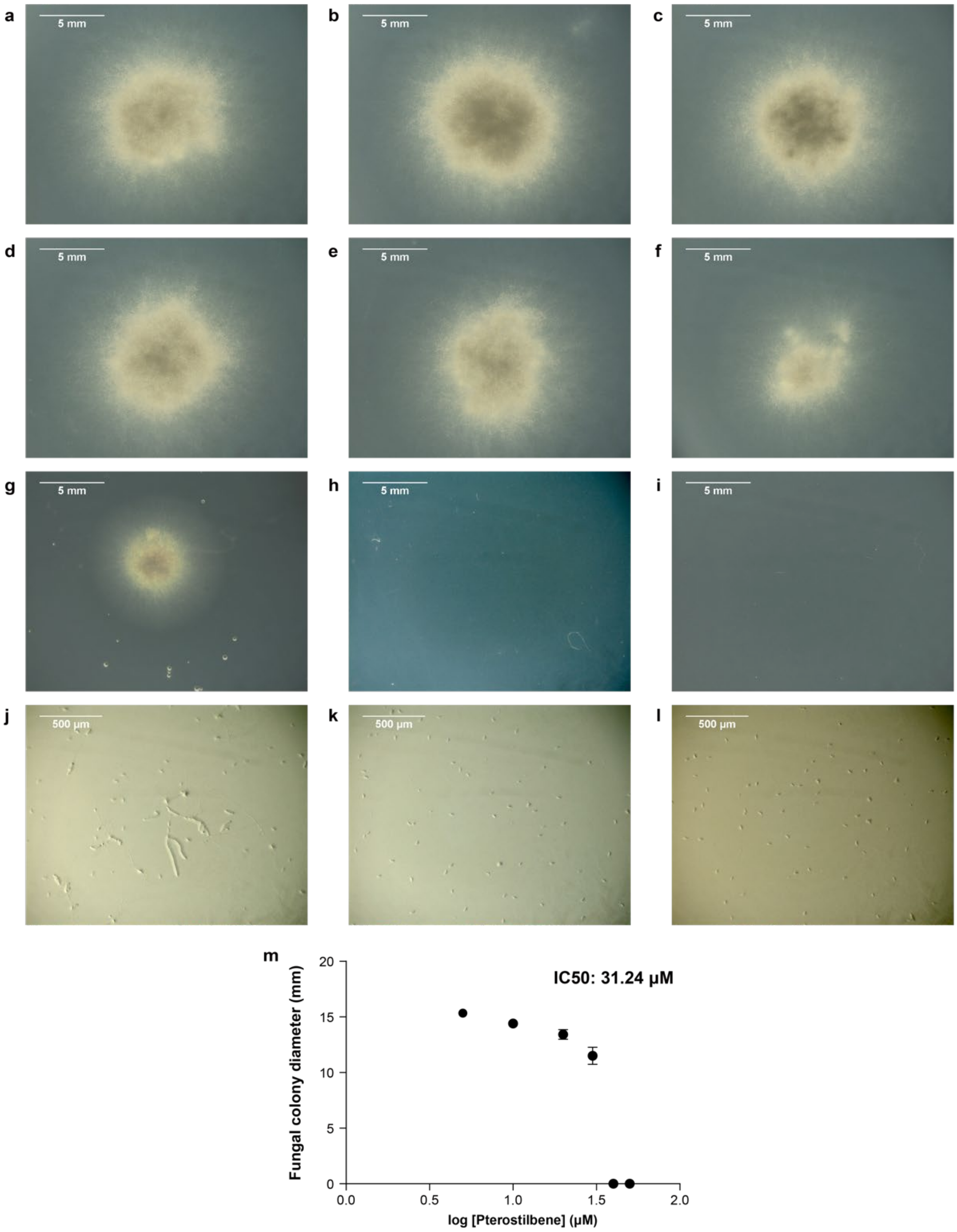

Supplementary Figure 20. *In vitro* toxicity of stilbenes towards *Colletotrichum sublineola*.

a–i, Mycelial growth of *C. sublineola* on potato dextrose agar after a 72-hour incubation with 4% (v/v) DMSO (**a**), 50 µM resveratrol (**b**), 50 µM pinostilbene (**c**), 5 µM pterostilbene (**d**), 10 µM pterostilbene (**e**), 20 µM pterostilbene (**f**), 30 µM pterostilbene (**g**), 40 µM pterostilbene (**h**) and 50 µM pterostilbene (**i**). Scale bars denote 5 mm.

j–l, Spore germination of *C. sublineola* on potato dextrose agar after a 12-hour

incubation in darkness with 4% (v/v) DMSO (**j**), 40 μM pterostilbene (**k**) and 50 μM pterostilbene (**l**). Scale bars denote 500 μm.

m, Diameter of *C. sublineola* colonies after a 72-hour incubation with different concentrations of pterostilbene. IC50 was estimated by GraphPad Prism 6. Values refer to means ± SD (n = 5).

IC50, half-maximal inhibitory concentration.

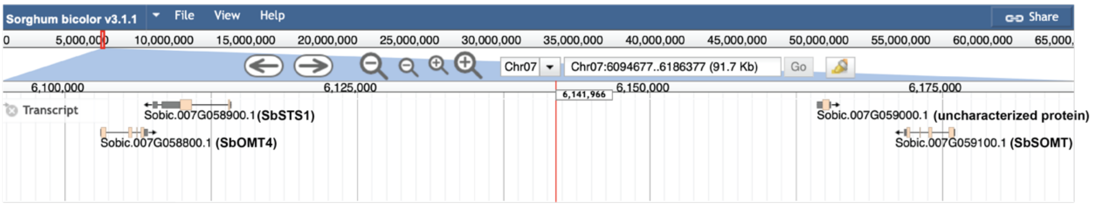

Supplementary Figure 21. Arrangement of *SbSTS1* (*Sb07g004700*; *Sobic.007G058900*), *SbOMT4* (*Sb07g004690*; *Sobic.007G058800*), and *SbSOMT* (*Sb07g004710*; *Sobic.007G059100*) in sorghum genome.

Gene arrangement in Chr07:6094677..6186377 of sorghum genome was obtained from Phytozome v13[65].

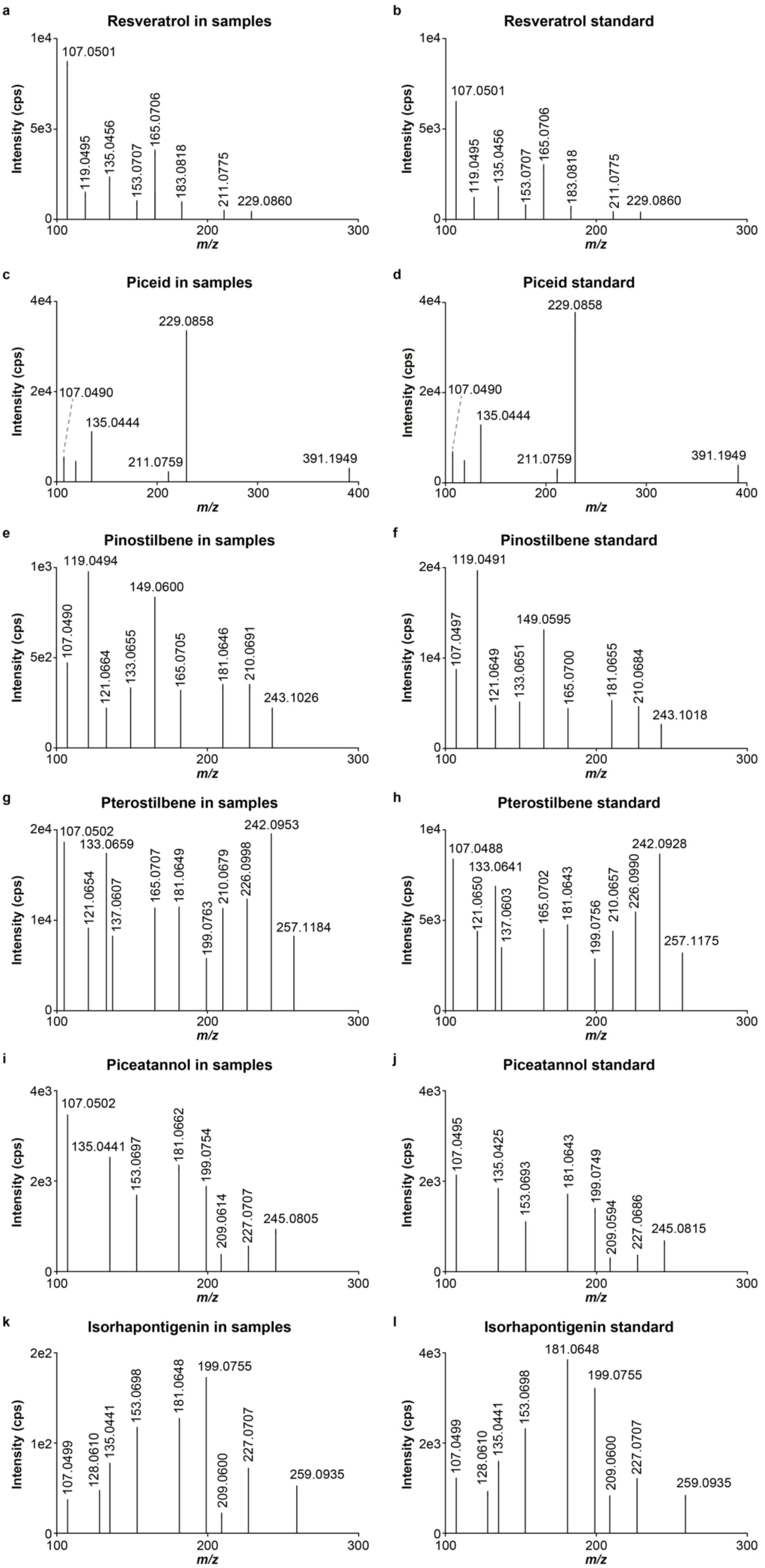
a
Resveratrol in samples
107.0501
119.0495
135.0456
153.0707
165.0706
183.0818
211.0775
229.0860
b
Resveratrol standard
107.0501
119.0495
135.0456
153.0707
165.0706
183.0818
211.0775
229.0860
c
Piceid in samples
229.0858
107.0490
135.0444
211.0759
391.1949
d
Piceid standard
229.0858
107.0490
135.0444
211.0759
391.1949
e
Pinostilbene in samples
119.0494
149.0600
107.0490
121.0664
133.0655
165.0705
181.0646
210.0691
243.1026
f
Pinostilbene standard
119.0491
107.0497
149.0595
121.0649
133.0651
165.0700
181.0655
210.0684
243.1018
g
Pterostilbene in samples
107.0502
133.0659
242.0953
121.0654
137.0607
165.0707
181.0649
199.0763
210.0679
226.0998
257.1184
h
Pterostilbene standard
107.0488
133.0641
242.0928
121.0650
137.0603
165.0702
181.0643
199.0756
210.0657
226.0990
257.1175
i
Piceatannol in samples
107.0502
135.0441
153.0697
181.0662
199.0754
209.0614
227.0707
245.0805
j
Piceatannol standard
107.0495
135.0425
153.0693
181.0643
199.0749
209.0594
227.0686
245.0815
k
Isorhapontigenin in samples
107.0499
128.0610
135.0441
153.0698
181.0648
199.0755
209.0600
227.0707
259.0935
l
Isorhapontigenin standard
181.0648
199.0755
107.0499
128.0610
135.0441
153.0698
209.0600
227.0707
259.0935
Intensity (cps)
m/z

Supplementary Figure 22. MSMS spectrum of stilbenes detected in plant samples and authentic standards.

a-b, Representative MSMS spectrum of resveratrol detected by HPLC-QTOF-HRMS in (**a**) *C. sublineola*-infected sorghum mesocotyls or mechanically-wounded wild sugarcane, and (**b**) authentic resveratrol standard.

c-d, Representative MSMS spectrum of piceid detected by HPLC-QTOF-HRMS in (**c**) *C. sublineola*-infected sorghum mesocotyls, and (**d**) authentic piceid standard.

e-f, Representative MSMS spectrum of pinostilbene detected by HPLC-QTOF-HRMS in (**e**) *C. sublineola*-infected sorghum mesocotyls, and (**f**) authentic pinostilbene standard.

g-h, Representative MSMS spectrum of pterostilbene detected by HPLC-QTOF-HRMS in (**g**) *C. sublineola*-infected sorghum mesocotyls, and (**h**) authentic pterostilbene standard.

i-j, Representative MSMS spectrum of piceatannol detected by HPLC-QTOF-HRMS in (**i**) mechanically-wounded wild sugarcane, and (**j**) authentic piceatannol standard.

k-l, Representative MSMS spectrum of isorhapontigenin detected by HPLC-QTOF-HRMS in (**k**) mechanically-wounded wild sugarcane, and (**l**) authentic isorhapontigenin standard.

cps, counts per second; *m/z*, mass-to-charge ratio. Source data are provided in the Source Data file.

Supplementary Table 1. High-performance liquid chromatography-quadrupole time-of-flight MS (HPLC-QTOF-MS) analysis of flavone aglycones in control or *Colletotrichum sublineola*-infected mesocotyls of resistant (SC748-5) and susceptible (BTx623) sorghum genotypes.

	BTx623		SC748-5	
	Control	Infected	Control	Infected
Time (h)	Apigenin [peak area (cps x 10^6) g^{-1} FW]			
24	4.08 ± 0.19	5.48 ± 4.58	3.95 ± 0.57	4.10 ± 0.30
48	7.18 ± 2.34	12.41 ± 1.67	8.06 ± 0.83	52.27 ± 12.73
72	12.25 ± 1.93	105.55 ± 31.98	12.24 ± 2.55	33.61 ± 9.98
96	13.11 ± 1.04	272.56 ± 100.65	7.42 ± 1.71	20.59 ± 3.92
	Luteolin [peak area (cps x 10^6) g^{-1} FW]			
24	1.20 ± 0.25	0.11 ± 0.05	2.38 ± 0.57	2.57 ± 0.45
48	1.16 ± 0.11	1.66 ± 0.43	3.37 ± 1.35	158.69 ± 29.51
72	1.62 ± 0.09	21.66 ± 10.97	4.94 ± 1.60	512.03 ± 65.46
96	5.35 ± 0.82	65.99 ± 32.20	7.03 ± 2.30	497.52 ± 135.58
	Chrysoeriol [peak area (cps x 10^6) g^{-1} FW]			
24	3.19 ± 2.60	4.66 ± 0.87	4.75 ± 1.15	8.88 ± 0.86
48	6.18 ± 0.27	8.02 ± 0.64	9.54 ± 1.11	108.23 ± 14.69
72	10.26 ± 1.15	28.49 ± 5.22	13.31 ± 1.97	308.20 ± 24.77
96	12.91 ± 1.07	75.03 ± 21.31	14.01 ± 4.62	364.55 ± 100.89
	Tricin [peak area (cps x 10^7) g^{-1} FW]			
24	2.58 ± 0.27	1.59 ± 1.17	41.14 ± 9.33	35.36 ± 2.76
48	2.75 ± 0.55	2.62 ± 0.20	59.66 ± 10.28	75.39 ± 16.11
72	3.80 ± 1.03	4.52 ± 0.53	88.92 ± 6.14	242.87 ± 31.12
96	4.58 ± 0.04	9.76 ± 1.42	73.18 ± 24.74	396.46 ± 22.25

Compound annotation was achieved by comparing the accurate mass and fragmentation pattern of individual peaks with those from authentic standards. Apigenin-d_5 was used as an internal standard for quantitation. Values refer to means ± SD (n = 3).

cps: count per second, FW: fresh weight.

Supplementary Table 2. High-performance liquid chromatography-quadrupole time-of-flight MS (HPLC-QTOF-MS) analysis of 3-deoxyanthocyanidin aglycones in mock or *Colletotrichum sublineola*-infected mesocotyls of resistant (SC748-5) and susceptible (BTx623) sorghum genotypes.

	BTx623		SC748-5	
	Control	Infected	Control	Infected
Time (h)	Apigeninidin [peak area (cps x 10^8) g^{-1} FW]			
48	n.d.	1.11 ± 0.25	n.d.	4.69 ± 1.65
72	n.d.	10.52 ± 5.66	n.d.	10.93 ± 1.72
96	n.d.	19.37 ± 2.09	n.d.	9.49 ± 1.24
	Luteolinidin [peak area (cps x 10^6) g^{-1} FW]			
48	n.d.	2.44 ± 0.65	n.d.	822.19 ± 271.66
72	n.d.	153.69 ± 94.92	n.d.	2735.93 ± 339.38
96	n.d.	246.27 ± 45.66	n.d.	2403.59 ± 761.50
	Diosmetinidin [peak area (cps x 10^6) g^{-1} FW]			
48	n.d.	0.97 ± 0.12	n.d.	167.38 ± 78.93
72	n.d.	21.13 ± 12.19	n.d.	803.91 ± 213.56
96	n.d.	46.81 ± 10.43	n.d.	1352.29 ± 185.15

Compound annotation was achieved by comparing the accurate mass and fragmentation pattern of individual peaks with those from authentic standards. Apigenin-d_5 was used as an internal standard for quantitation. Values refer to means ± SD (n = 3).

cps: counts per second, FW: fresh weight, n.d.: not detected.

Supplementary Table 3. High-performance liquid chromatography-quadrupole time-of-flight MS (HPLC-QTOF-MS) analysis of stilbenes in mock or *Colletotrichum sublineola*-infected mesocotyls of resistant (SC748-5) and susceptible (BTx623) sorghum genotypes.

	BTx623		SC748-5	
	Control	Infected	Control	Infected
Time (h)	Resveratrol (μg g^{-1} FW)			
72	n.d.	0.03 ± 0.01	n.d.	0.31 ± 0.00
96	n.d.	0.36 ± 0.10	n.d.	0.91 ± 0.19
	Piceid (μg g^{-1} FW)			
72	n.d.	2.63 ± 0.24	n.d.	8.00 ± 0.46
96	n.d.	6.83 ± 0.75	n.d.	19.62 ± 3.24
	Pinostilbene (μg g^{-1} FW)			
72	n.d.	0.05 ± 0.00	n.d.	0.22 ± 0.06
96	n.d.	0.08 ± 0.02	n.d.	0.57 ± 0.14
	Pterostilbene (μg g^{-1} FW)			
48	n.d.	n.d.	n.d.	0.03 ± 0.02
72	n.d.	0.16 ± 0.06	n.d.	1.56 ± 0.12
96	n.d.	0.71 ± 0.15	n.d.	5.65 ± 1.89

Compound annotation was achieved by comparing the accurate mass and fragmentation pattern of individual peaks with those from authentic standards. Apigenin-d_5 was used as an internal standard for quantitation. Values refer to means ± SD (n = 3).

FW: fresh weight, n.d.: not detected.

Supplementary Table 4. High-performance liquid chromatography-quadrupole time-of-flight MS (HPLC-QTOF-MS) analysis of stilbenes in mock or *Colletotrichum sublineola*-infected mesocotyls of wild-type Tx430 and *sbsomt* mutants.

	Tx430	*sbsomt-a*	*sbsomt-b1*	*sbsomt-b2*
Time (h)	Resveratrol (μg g^{-1} FW)			
72	0.09 ± 0.12	0.09 ± 0.02	0.10 ± 0.03	0.11 ± 0.04
96	0.18 ± 0.03	0.31 ± 0.12	0.49 ± 0.07	0.53 ± 0.03
	Piceid (μg g^{-1} FW)			
72	1.60 ± 0.25	2.60 ± 1.20	1.78 ± 1.06	1.97 ± 1.34
96	5.74 ± 0.70	8.03 ± 2.33	18.14 ± 2.32	17.14 ± 5.45
	Pinostilbene (μg g^{-1} FW)			
72	0.03 ± 0.00	n.d.	n.d.	n.d.
96	0.09 ± 0.02	n.d.	n.d.	n.d.
	Pterostilbene (μg g^{-1} FW)			
72	0.13 ± 0.02	n.d.	n.d.	n.d.
96	1.84 ± 0.32	n.d.	n.d.	n.d.

Compound annotation was achieved by comparing the accurate mass and fragmentation pattern of individual peaks with those from authentic standards. Apigenin-d_5 was used as an internal standard for quantitation. Values refer to means ± SD (n = 3).

FW: fresh weight, n.d.: not detected.

Supplementary Table 5. Dissociation constant (K_d) of SbSOMT mutants with stilbenes

Stilbene	SbSOMT mutant	Stoichiometry, N	K_d (μM)
Resveratrol	I144N	0.6	151.6 ± 31.1
	D283A	0.58 ± 0.01	1.31 ± 0.13
	E310A	0.36 ± 0.04	18.5 ± 2.58
	F337N	-	No binding
	E342A	0.75 ± 0.01	11.1 ± 0.75
	I144N/F337N	-	No binding
	H282N/D283A	0.61 ± 0.17	32.90 ± 5.93
Pinostilbene	E310A	0.59 ± 0.01	3.91 ± 0.33
Pterostilbene	E310A	0.40 ± 0.08	6.04 ± 2.08

Results are expressed as means of K_d ± SE derived from curve fitting.

N of I144N was fixed at 0.6 (average to reported Ns in this table) to estimate the K_d.

Supplementary Table 6. High-performance liquid chromatography-quadrupole time-of-flight MS (HPLC-QTOF-MS) analysis of stilbenes in mechanically-wounded stalks wild sugarcane.

	Wounded wild sugarcane		
Time (h)	Resveratrol ($\mu g\ g^{-1}$ FW)	Piceatannol ($\mu g\ g^{-1}$ FW)	Isorhapontigenin ($\mu g\ g^{-1}$ FW)
0	n.d.	n.d.	n.d.
24	n.d.	n.d.	n.d.
72	2.55 ± 1.55	9.37 ± 5.98	1.03 ± 0.87
120	7.87 ± 3.03	33.79 ± 4.50	2.18 ± 1.77

Compound annotation was achieved by comparing the accurate mass and fragmentation pattern of individual peaks with those from authentic standards. Apigenin-d_5 was used as an internal standard for quantitation. Values refer to means ± SD (n = 3).

FW: fresh weight, n.d.: not detected.

Supplementary Table 7. Dissociation constants of SsCOMT with stilbenes.

	K_d (µM)
	SsCOMT
Resveratrol	2.78 ± 0.33
Pinostilbene	5.57 ± 0.77
Pterostilbene	9.93 ± 4.04
Piceatannol	25.50 ± 14.20
SAM	25.00 ± 12.90

Results are expressed as means of K_d ± standard error derived from curve fitting.

Supplementary Table 8. List of OMT-ligand complexes deposited to PDB and the equivalent residues of SbCOMT^{Asn128}/ SbSOMT^{Ile144} and SbCOMT^{Asn323}/SbSOMT^{Phe337}.

PDB ID	UNIPROT	Residue at equivalent positions		Ligand identity	Vicinal functional group# and polarity	Reference
		SbCOMT^{Asn128}/ SbSOMT^{Ile144}	SbCOMT^{Asn323}/ SbSOMT^{Phe337}			
1FP1	P93324	Leu139	Val333	Substrate	-H and non-polar	[43]
1KYW	P28002	Asn131	Asn324	Substrate	-OH and polar	[44]
1KYZ	P28002	Asn131	Asn324	Product	-OH and polar	
1ZG3	Q29U70	Ala126	Phe326	Substrate	-H and non-polar	[54]
1ZGA	Q29U70	Ala126	Phe326	Substrate	-H and non-polar	
1ZGJ	Q29U70	Ala126	Phe326	Product	-H and non-polar	
2QYO	Q06YR3	Val123	Val319	Canonical product	-H and non-polar	NA
3I58	Q84HC8	Asp107	Phe294	Product	-H and non-polar	[55]
3I5U	Q84HC8	Asp107	Phe294	Substrate analogue (non-reactive)	Not compared	
3I64	Q84HC8	Asp107	Phe294	Substrate analogue (reactive)	Fused benzene ring and non-polar	
3P9I	Q9ZTU2	Asn128	Asn321	Product	-OH and polar	[22]

Supplementary Table 8. List of OMT-ligand complexes deposited to PDB and the equivalent residues of SbCOMT^{Asn128}/ SbSOMT^{Ile144} and SbCOMT^{Asn323}/SbSOMT^{Phe337} (continued).

3P9K	Q9ZTU2	Asn128	Asn321	Product	-OH and polar	[22]
3REO	O04385	Ala134	Asn327	Substrate	-OMe and polar	[56]
3TKY	O04385	Ala134	Asn327	Substrate	-OMe and polar	
5CVU	O04385	Ala134	Asn327	Substrate	-OMe and polar	
5I2H	D5STZ7	Ala110	Thr322	Non-productive conformation	Not compared	NA
5ICE	Q5C9L7	Ile114	Thr311	Substrate	-H and non-polar	[44]
5ICF	Q5C9L7	Ile114	Thr311	Non-reactive inhibitor	Not compared	
5XOH	A0A166U5H3	His126	Val320	Substrate	Fused furan ring and non-polar	[57]
6I6L	I3V6A7	Thr157	Asn351	Product	-OMe and polar	[46]
6I6M	I3V6A7	Thr157	Asn351	Substrate	-OMe and polar	
6I72	Q9M602	Asn131	Asn324	Canonical substrate	-OH and polar	NA
6I73	Q9M602	Asn131	Asn324	Canonical substrate	-OH and polar	
6NEJ	Q5C9L2	Thr115	Asn309	Canonical substrate	-OMe and polar	NA

[#]The vicinal functional group refers to the functional group vicinal to the reactive site positioned between catalytic dyads and SbCOMT^{Asn323}/ SbSOMT^{Phe337} equivalent position as depicted in Supplementary Fig. **18-19**. NA, Not available (work to be published).

Supplementary Table 9. **Primer used in this study.**

Primer name	Sequence (5' to 3') (restriction sites added are underlined)	Purposes
SbEIF4α-qRT-F	CAACTTTGTCACCCGCGATGA	qRT-PCR experiments for *SbEIF4α*
SbEIF4α-qRT-R	TCCAGAAACCTTAGCAGCCCA	
SbSTS-qRT-F	TGCTACGGTGTTGGCCATT	qRT-PCR experiments for *SbSTS1*
SbSTS-qRT-R	ATCGACTTGTGGCATATCCTCTT	
SbOMT4-qRT-F	CCTAAAGTGGATTCTTCACGATTG	qRT-PCR experiments for *SbOMT4*
SbOMT4-qRT-R	GCTAGTTGCGATGTTAGTGTTTC	
SbSOMT-qRT-F	AGGTGGCAGCATGTTCGATAA	qRT-PCR experiments for *SbSOMT*
SbSOMT-qRT-R	ACGATCACCTTGCCTCTCAC	
SsGADPH-qRT-F	TTGGTTTCCACTGACTTCGTT	qRT-PCR experiments for *SsGADPH*
SsGADPH-qRT-R	CTGTAGCCCCACTCGTTGT	
SsSTS-qRT-F	AGAGCGAACACCTTACCGAC	qRT-PCR experiments for *SsSTS*
SsSTS-qRT-R	GCCGAGTACGAGCTCATGTT	
SsCOMT-qRT-F	GAGGACAAGGACGGCAAGTA	qRT-PCR experiments for *SsCOMT*
SsCOMT-qRT-R	ACCGCGTCCTTGAGGTAGTA	
SbOMT4-RE-F	CGCGGATCCATGGGCAGCTATACTACCAG	Cloning of *SbOMT4* in pET23a(+) vector for recombinant protein expression in *E. coli*
SbOMT4-RE-R	CCCAAGCTTCTTTGTGAATTCAAGGGCCC	
SbSOMT-HF-F	TGAGAACCTGTACTTCCAAGGCAGCTACGACAGCAGCAGTAG	HiFi Assembly of *SbSOMT* (2-377) in pET-N-His-TEV vector for recombinant protein expression in *E. coli*
SbSOMT-HF-R	AGCCGGATCTCACTCGAGTTACTTTGTGAACTCAAGGGCCCAGAC	

Supplementary Table 9. **Primer used in this study (continued).**

Primer name	Sequence (5' to 3') (restriction sites added are underlined)	Purposes
SbCOMT-HF-F	TGAGAACCTGTACTTCCAAGGGTCGACGGCGGAGGAC	HiFi Assembly of *SbCOMT* (2-362) in pET-N-His-TEV vector for recombinant protein expression in *E. coli*
SbCOMT-HF-R	AGCCGGATCTCACTCGAGTTACTTGATGAACTCGATGGCCCAG	
SsSTS-Gib-F	CTTCTGCAGGAATTCGATATCATGACTGGGAAGGTAACATTGGGG	Gibson Assembly of *SsSTS* in pET-N-His-TEV vector for recombinant protein expression in *E. coli*
SsSTS-Gib-R	GAGAGATCTGTCGACGATATCCTACACTGTGATGATGGGAACGC	
SsCOMT-Gib-F	CTTCTGCAGGAATTCGATATCATGGGCTCGACCGCC	Gibson Assembly of *SsCOMT* in pET-N-His-TEV vector for recombinant protein expression in *E. coli*
SsCOMT-Gib-R	GAGAGATCTGTCGACGATATCTTACTTGATGAACTCGATGGCCCAG	
SbSOMT-I144N-F	GTGCTTCCGCTTGGGATGATGAACCTAAACAAGACATTCCTGGACAGC	Generation of SbSOMT I144N and I144N/F337N mutant proteins
SbSOMT-I144N-R	CATCATCCCAAGCGGAAGCAC	
SbSOMT-F337N-F	CCTCACCATGCTGGTCACGAACGGCAGTGGTAAAGAGAGGACACA	Generation of SbSOMT F337N and I144N/F337N mutant proteins
SbSOMT-F337N-R	CGTGACCAGCATGGTGAGG	
SbSOMT-H282A-F	TTCTGCTCAAGTGGATTCTTGCTGATTGGGACGACAAGGCGTG	Generation of SbSOMT H282A mutant protein
SbSOMT-H282A-R	AAGAATCCACTTGAGCAGAACTGCA	
SbSOMT-H282N-F	GATTGGGACGACAAGGCGTG	Generation of SbSOMT H282N mutant protein
SbSOMT-H282N-R	GTTAAGAATCCACTTGAGCAGAAC	Generation of SbSOMT H282N and H282N/D283A mutant proteins
SbSOMT-D283A-F	GCGTGGGACGACAAGG	Generation of SbSOMT D283A and H282N/D283A mutant proteins
SbSOMT-D283A-R	ATGAAGAATCCACTTGAGCAGAAC	Generation of SbSOMT D283A mutant protein

Supplementary Table 9. **Primer used in this study (continued).**

Primer name	Sequence (5' to 3') (restriction sites added are underlined)	Purposes
SbSOMT-E310A-F	GCGTACGTTGTTCCGGATG	Generation of SbSOMT E310A mutant protein
SbSOMT-E310A-R	CAGAACGATCACCTTGCCTC	
SbSOMT-E342A-F	GCGAGGACACAGAGGGAGTTC	Generation of SbSOMT E342A mutant protein
SbSOMT-E342A-R	TTTACCACTGCCAAACGTGAC	
sbsomt-gPCR-F1	TGACTCATCAGCACGGAACG	Genotyping of *sbsomt* CRISPR/Cas9 mutants
sbsomt-gPCR-R1	CACCCTCCTAGTAGCCGCAA	
sbsomt-gPCR-F2	GGCAAAACATTACGGATGCAGT	Genotyping of *sbsomt* CRISPR/Cas9 mutants
sbsomt-gPCR-R2	TGACTTCACGGAGGCTCATAG	
SbSTS-pEAQ-F	ATCGGACCGGTATGACGACTGGGAAGGTAAC	Cloning of *SbSTS1* in pEAQ-HT vector for *N. benthamiana* expression
SbSTS-pEAQ-R	CGATCCTCGAGTCATGCAGCCACTGTGGTGA	
SbSOMT-pEAQ-F	TGCCCAAATTCGCGAATGGGCAGCTACGACAGC	Cloning of *SbSOMT* in pEAQ-HT vector for *N. benthamiana* expression
SbSOMT-pEAQ-R	TGGTGATGGTGATGCATATTATTTTTCAAATTGAGGATGAGACCACTTT GTGAACTCAAGGGCCCAG	

Supplementary Table 10. **Protein and ligand concentrations used for ITC setup.**

Protein in cell	Protein concentration (µM)	Ligand in syringe	Ligand concentration (µM)
SbSOMT, WT	50	Resveratrol	750
	50	Pinostilbene	750
	25	Pterostilbene	500
	50	Piceatannol	1000
	50	SAM	2000
SbCOMT, WT	50	Resveratrol	750
	50	Pinostilbene	750
	25	Pterostilbene	500
	50	Piceatannol	750
	50	SAM	1500
SbSOMT mutants:			
I144N	25	Resveratrol	1000
D283A	50	Resveratrol	1000
E310A	50	Resveratrol	1000
	50	Pinostilbene	1000
	25	Pterostilbene	500
F337N	25	Resveratrol	1000
E342A	50	Resveratrol	1000
I144N/F337N	25	Resveratrol	1000
H282N/D283A	25	Resveratrol	1000
SsCOMT, WT	50	Resveratrol	750
	50	Pinostilbene	750
	25	Pterostilbene	500
	50	Piceatannol	1000
	50	SAM	2000

Supplementary Table 11. **X-ray data collection and refinement statistics of structures reported in this study.**

	SbSOMT-Resveratrol-β-NAD	SbSOMT-Pinostilbene-β-NAD	SbSOMT-Pterostilbene-β-NAD	SbSOMT-Resveratrol
**Data collection**				
Wavelength (Å)	0.9785	0.9784	0.9784	0.9785
Resolution (Å) [a]	48.42 – 1.72 (1.76 – 1.72)	19.70 – 2.10 (2.14 – 2.10)	31.40 – 2.40 (2.44 – 2.40)	48.91 – 2.56 (2.65 – 2.56)
Space group	$P\ 3_1\ 2\ 1$	$P\ 3_1\ 2\ 1$	$P\ 3_1\ 2\ 1$	$P\ 2_1\ 2_1\ 2_1$
**Cell dimensions**				
a, b, c (Å)	96.8, 96.8, 168.0	96.7, 96.7, 166.9	96.5, 96.5, 169.4	97.5, 111.7, 131.1
α, β, γ (°)	90, 90, 120	90, 90, 120	90, 90, 120	90, 90, 90
Total reflections	1882940	651200	709365	616499
Unique reflections	97225	101522	36363	46864
Multiplicity	19.4	6.4	19.5	13.2
Completeness (%) [a]	99.8 (97.1)	99.8 (99.0)	99.2 (99.9)	99.9 (100.0)
Mean I/sigma (I) [a]	30.2 (2.9)	9.2 (2.0)	7.5 (0.8)	16.3 (2.4)
Wilson B-factor (Å^2)	22.6	31.43	36.78	52.2
R_{merge} (%)[a]	6.0 (94.1)	21.8 (72.4)	19.9 (95.5)	11.8 (115.6)
R_{meas} (%)[a]	6.3 (101.0)	23.8 (78.8)	20.4 (98.2)	12.7 (124.7)
R_{pim} (%)[a]	2.0 (35.7)	9.4 (31.0)	4.6 (22.8)	4.8 (46.8)
$CC_{1/2}$ [a]	1.000 (0.849)	0.891 (0.790)	0.915 (0.871)	0.999 (0.902)

[a] Value relative to the highest resolution shell are given in parentheses.

Supplementary Table 11. **X-ray data collection and refinement statistics of structures reported in this study (continued).**

	SbSOMT-Resveratrol-β-NAD	SbSOMT-Pinostilbene-β-NAD	SbSOMT-Pterostilbene-β-NAD	SbSOMT-Resveratrol
**Refinement**				
Reflections used in refinement	97159	53133	36012	46802
Reflections used for R-free	4899	2652	1899	2368
R-work	0.163	0.171	0.187	0.182
R-free	0.190	0.202	0.246	0.252
Number of atoms	6638	6216	6104	11441
Macromolecules	5841	5674	5721	11286
Ligands/Ions	229	274	214	92
Solvent	568	268	169	63
rmsd [b] bond lengths (Å)	0.012	0.015	0.016	0.016
rmsd [b] bond angles (°)	1.73	2.023	2.10	2.22
**Ramachandran plot**				
Favored (%)	99.03	97.36	95.57	95.72
Allowed (%)	0.97	2.22	3.60	3.31
Outlier (%)	0.00	0.42	0.83	0.97
Sidechain outlier (%)	0.64	2.64	1.96	4.56
Molprobity Clash score	4.46	8.69	7.95	8.10
Mean B-value (Å^2)	29.0	37.0	46.0	68.0
Macromolecules	28.38	36.72	45.54	62.15
Ligands/Ions [c]	53.31 (41.01)	61.19	78.06	52.78
Solvent	36.14	39.74	43.73	41.98
**PDB ID code**	**7VB8**	**7WAR**	**7WAS**	**7WAQ**

[b] rmsd, root mean square deviation.

[c] The mean B-values (Å^2) of ligands and ions are given separately, with value for ion given in parentheses.

Supplementary Table 12. Accession numbers of sequences used in this study.

Protein	GenBank accession number
Arabidopsis thaliana AtOMT1	NP_200227
Carthamus tinctorius CtCAldOMT	BAG71895
Catharanthus roseus CrCOMT	Q8W013
Hordem vulgare HvCOMT	ABQ58825
Medicago sativa MsCOMT	P28002
Medicago sativa MsIOMT	AAC49927
Oryza sativa OsCAldOMT1	Q6ZD89
Oryza sativa OsNOMT	Q0IP69
Panicum virgatum PvCOMT	ADX98508
Pinus sylvestris PsPMT2	AQX17823
Pinus taeda PtAEOMT	Q43096
Populus tremuloides PtCOMT	AAB61731
Rosa hybrid RhOOMT	AAM23004
Solanum lycopersicum SlCOMT	XP_004235028
Sorghum bicolor SbCOMT	AAO43609
Sorghum bicolor SbNOMT	XP_002465645
Sorghum bicolor SbOMT1	ABP01563
Sorghum bicolor SbOMT3	ABP01564
Sorghum bicolor SbOMT4	XP_002443937
Sorghum bicolor SbSOMT	XP_021320201
Sorghum bicolor SbSTS1	XP_002445139
Triticum aestivum TaCOMT1	AAP23942
Triticum aestivum TaCOMT2	Q38J50
Vitis vinifera VvCOMT	NP_001268100
Vitis vinifera VvROMT	CAQ76879
Zea mays ZmCOMT	Q06509
Zea mays ZmNOMT	XP_020399799
**Gene**	**Ensembl Plants Identifier**
Saccharum spontaneum SsSTS	Sspon.06G0010290-2P
Saccharum spontaneum SsCOMT	Sspon.06G0010980-3C
**Gene**	**GenBank accession number**
Sorghum halepense ShOMT1	GGDZ01007645
Sorghum halepense ShSOMT	GGDZ01140964
