# Supplementary material for: Regioselective stilbene O-methylations in Saccharinae grasses
Source: Nat Commun. 2023 Jun 12;14:3462. doi: 10.1038/s41467-023-38908-5 (PMC10261104; doi:10.1038/s41467-023-38908-5)
Supplement: Supplementary file 3 — Reporting Summary [file 41467_2023_38908_MOESM3_ESM.pdf]

nature portfolio

Corresponding author(s): Quan Hao & Clive Lo

Last updated by author(s): May 8, 2023

Reporting Summary

Nature Portfolio wishes to improve the reproducibility of the work that we publish. This form provides structure for consistency and transparency in reporting. For further information on Nature Portfolio policies, see our Editorial Policies and the Editorial Policy Checklist.

Statistics

For all statistical analyses, confirm that the following items are present in the figure legend, table legend, main text, or Methods section.

n/a	Confirmed	
☐	☒	The exact sample size (*n*) for each experimental group/condition, given as a discrete number and unit of measurement
☐	☒	A statement on whether measurements were taken from distinct samples or whether the same sample was measured repeatedly
☐	☒	The statistical test(s) used AND whether they are one- or two-sided<br>*Only common tests should be described solely by name; describe more complex techniques in the Methods section.*
☒	☐	A description of all covariates tested
☐	☒	A description of any assumptions or corrections, such as tests of normality and adjustment for multiple comparisons
☐	☒	A full description of the statistical parameters including central tendency (e.g. means) or other basic estimates (e.g. regression coefficient) AND variation (e.g. standard deviation) or associated estimates of uncertainty (e.g. confidence intervals)
☐	☒	For null hypothesis testing, the test statistic (e.g. *F*, *t*, *r*) with confidence intervals, effect sizes, degrees of freedom and *P* value noted<br>*Give P values as exact values whenever suitable.*
☒	☐	For Bayesian analysis, information on the choice of priors and Markov chain Monte Carlo settings
☒	☐	For hierarchical and complex designs, identification of the appropriate level for tests and full reporting of outcomes
☒	☐	Estimates of effect sizes (e.g. Cohen's *d*, Pearson's *r*), indicating how they were calculated

Our web collection on statistics for biologists contains articles on many of the points above.

Software and code

Policy information about availability of computer code

Data collection	SCIEX OS and Analyst software version 1.5.6 were used to collect LCMS data collected from X500r and 3200QTRAP mass spectrometers, respectively.
Data analysis	SCIEX OS and Analyst software version 1.5.6 were used to analyze LCMS data collected from X500r and 3200QTRAP mass spectrometers, respectively. X-ray diffraction data were processed by either XDS or HKL3000 package. Data were analysed using pipelines provided in CCP4 suite with details described in the Methods section. Figures featuring protein structure were prepared using UCSF Chimera or UCSF ChimeraX. Ligand docking was conducted using AutoDock Vina. ImageJ software was used to quantify fungal colony size and Graphpad Prism 6 was used to process corresponding data.

For manuscripts utilizing custom algorithms or software that are central to the research but not yet described in published literature, software must be made available to editors and reviewers. We strongly encourage code deposition in a community repository (e.g. GitHub). See the Nature Portfolio guidelines for submitting code & software for further information.

Data

Policy information about availability of data

All manuscripts must include a data availability statement. This statement should provide the following information, where applicable:

- Accession codes, unique identifiers, or web links for publicly available datasets
- A description of any restrictions on data availability
- For clinical datasets or third party data, please ensure that the statement adheres to our policy

Unprocessed metabolic data (including LC traces and MS/MS spectra) of sorghum and wild sugarcane are provided in the Source Data file. Raw LC-MS data are available from the corresponding author (clivelo@hku.hk) upon request. Atomic coordinates and structure factors for the crystal structures reported in this work were deposited to the Protein Data Bank under accession numbers: 7VB8 [https://www.rcsb.org/structure/7VB8], 7WAQ [https://www.rcsb.org/structure/7WAQ], 7WAR [https://www.rcsb.org/structure/7WAR] and 7WAS [https://www.rcsb.org/structure/7WAS]. Source data are provided with this paper.

Research involving human participants, their data, or biological material

Policy information about studies with human participants or human data. See also policy information about sex, gender (identity/presentation), and sexual orientation and race, ethnicity and racism.

Reporting on sex and gender	Not applicable
Reporting on race, ethnicity, or other socially relevant groupings	Not applicable
Population characteristics	Not applicable
Recruitment	Not applicable
Ethics oversight	Not applicable

Note that full information on the approval of the study protocol must also be provided in the manuscript.

Field-specific reporting

Please select the one below that is the best fit for your research. If you are not sure, read the appropriate sections before making your selection.

☒ Life sciences ☐ Behavioural & social sciences ☐ Ecological, evolutionary & environmental sciences

For a reference copy of the document with all sections, see nature.com/documents/nr-reporting-summary-flat.pdf

Life sciences study design

All studies must disclose on these points even when the disclosure is negative.

Sample size	Three independent biological replicates were used for all experiments involving plant materials.
Data exclusions	No data was excluded.
Replication	All experiments were repeated at least two times independently with consistent trends and observations.
Randomization	Plants were separated into control or treatment group, and all samples were then collected based on experimental designs. No randomization was needed.
Blinding	Plants were separated into control or treatment group, and all samples were then collected based on experimental designs. No blinding was needed.

Reporting for specific materials, systems and methods

We require information from authors about some types of materials, experimental systems and methods used in many studies. Here, indicate whether each material, system or method listed is relevant to your study. If you are not sure if a list item applies to your research, read the appropriate section before selecting a response.

Materials & experimental systems

n/a	Involved in the study
[x]	[ ] Antibodies
[x]	[ ] Eukaryotic cell lines
[x]	[ ] Palaeontology and archaeology
[x]	[ ] Animals and other organisms
[x]	[ ] Clinical data
[x]	[ ] Dual use research of concern
[ ]	[x] Plants

Methods

n/a	Involved in the study
[x]	[ ] ChIP-seq
[x]	[ ] Flow cytometry
[x]	[ ] MRI-based neuroimaging

Dual use research of concern

Policy information about dual use research of concern

Hazards

Could the accidental, deliberate or reckless misuse of agents or technologies generated in the work, or the application of information presented in the manuscript, pose a threat to:

No	Yes
[x]	[ ] Public health
[x]	[ ] National security
[x]	[ ] Crops and/or livestock
[x]	[ ] Ecosystems
[x]	[ ] Any other significant area

Experiments of concern

Does the work involve any of these experiments of concern:

No	Yes
[x]	[ ] Demonstrate how to render a vaccine ineffective
[x]	[ ] Confer resistance to therapeutically useful antibiotics or antiviral agents
[x]	[ ] Enhance the virulence of a pathogen or render a nonpathogen virulent
[x]	[ ] Increase transmissibility of a pathogen
[x]	[ ] Alter the host range of a pathogen
[x]	[ ] Enable evasion of diagnostic/detection modalities
[x]	[ ] Enable the weaponization of a biological agent or toxin
[x]	[ ] Any other potentially harmful combination of experiments and agents
